# RANKL signaling in bone marrow mesenchymal stem cells negatively regulates osteoblastic bone formation

**DOI:** 10.1038/s41413-018-0035-6

**Published:** 2018-11-27

**Authors:** Xiao Chen, Xin Zhi, Jun Wang, Jiacan Su

**Affiliations:** 1 Department of Orthopedics Trauma, Shanghai Changhai Hospital, Second Military Medical University, Shanghai, China; 2 Department of Chemistry, Fudan University, Shanghai, China; 3 School of Basic Medical Sciences, Second Military Medical University, Shanghai, China; 4 College of Life Science, Fudan University, Shanghai, China

## Abstract

RANKL signaling is essential for osteoclastogenesis. Its role in osteoblastic differentiation and bone formation is unknown. Here we demonstrate that RANK is expressed at an early stage of bone marrow mesenchymal stem cells (BMSCs) during osteogenic differentiation in both mice and human and decreased rapidly. RANKL signaling inhibits osteogenesis by promoting β-catenin degradation and inhibiting its synthesis. In contrast, RANKL signaling has no significant effects on adipogenesis of BMSCs. Interestingly, conditional knockout of *rank* in BMSCs with *Prx1*-Cre mice leads to a higher bone mass and increased trabecular bone formation independent of osteoclasts. In addition, *rank*^flox/flox^: *Prx1*-Cre mice show resistance to ovariectomy-(OVX) induced bone loss. Thus, our results reveal that RANKL signaling regulates both osteoclasts and osteoblasts by inhibition of osteogenic differentiation of BMSCs and promotion of osteoclastogenesis.

## Developmental Genetics: A dual-use gene regulates bone formation and breakdown

Researchers in China have shown that a gene known for breaking bones down is also involved in making new bone. Bones are constantly repaired and reshaped by cells which break down bone tissue, osteoclasts, and those which create new bone, osteoblasts. The RANKL gene is known to play an important role in osteoclast development, but Jiacan Su of the Second Military Medical University has shown that it is also important for osteoblasts. Su’s team detected high expression of RANKL and its receptor, RANK, in bone marrow stem cells, and the levels decreased as the stem cells differentiated into osteoblasts. Artificially increasing RANK expression decreased osteoblast differentiation, while reducing its expression increased osteoblast differentiation and bone mass. By showing that RANKL regulates osteoblasts as well as osteoclasts, these findings open new avenues for understanding bones development.

## Introduction

Postnatal skeletal growth and bone remodeling are highly coordinated processes primarily mediated by bone-forming osteoblasts and bone-resorbing osteoclasts.^1–4^ Receptor activator of nuclear factor κ B Ligand (RANKL) signaling is vital for osteoclasts proliferation and differentiation.^5–7^ RANK knockout in mice leads to severe osteopetrosis due to the absence of mature osteoclasts.^7,8^ Up to now, RANKL signaling has been mostly investigated in osteoclastogenesis and its inhibitor denosumab has been introduced clinically in the treatment of osteoclasts-related diseases like osteoporosis.^9–13^ However, its roles in osteoblastic differentiation and postnatal bone formation have not been clarified. A recent study indicates that RANKL reverse signaling promotes osteoblastic differentiation,^14^ which shows that RANKL signaling could play an important role in bone formation.

RANK is over-expressed on osteosarcoma,^15,16^ a mesenchymal tumor with the osteoblastic origin and regulates osteoblast migration,^16^ essential to bone modeling and remodeling. The current studies imply that RANKL signaling could play a key role in osteoblastic differentiation and bone formation, which has long been underestimated.

In this study, we found that RANK was expressed at the early stage of osteogenic differentiation of bone marrow mesenchymal stem cells (BMSCs) and was downregulated after the osteogenic differentiation began. RANK inhibited osteoblastic differentiation of BMSCs while had no effects on adipogenesis. BMSCs conditional knockout of RANK with *Prx1*-Cre mice led to a significantly increased trabecular bone mass, accelerated bone formation rate and showed resistance to ovariectomy-(OVX) induced bone loss. Our results show that RANKL signaling regulates both osteoclasts and osteoblasts by inhibiting BMSCs osteogenic differentiation and promoting osteoclastogenesis.

## Results

### RANK is expressed in BMSCs

**Fig. 1:**
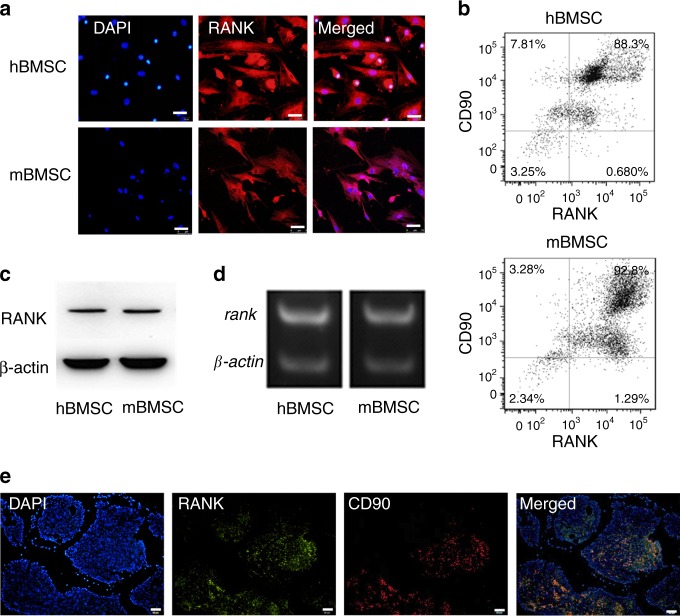
BMSCs express RANK. **a** Both human (h) and mouse (m) BMSCs express RANK assessed by immunofluorescence staining. Scale bar = 20 μm. **b** Flow cytometry confirmed human and mouse BMSCs express RANK. **c** and **d** Western blot, RT-PCR analysis. **e** Immunofluorescence shows that RANK is expressed in BMSCs in vivo. Scale bar = 50 μm

Although RANK is expressed in several osteosarcoma cell lines, its expression in BMSCs has not been determined. We hypothesized that RANK was expressed in BMSCs. We collected bone marrow from patients undergoing fracture surgeries and isolated human BMSCs identified by CD45^−^/Stro-1^+^/CD146^+^. We collected BMSCs from 6-week-old male mice identified by CD34^−^/CD45^−^/CD73^+^/CD90^+^/CD105^+^ (Figure S1). After passage, BMSCs of the third generation were used. We found that both human and mouse BMSCs highly expressed RANK in the cytoplasm, as assessed by immunofluorescence (Fig. 1a). Flow cytometric analysis showed that RANK and CD90 were co-expressed in 88.3% and 92.8% of human and mice BMSCS, respectively (Fig. 1b). Western blot and RT-PCR confirmed the RANK expressions in human and mouse BMSCs (Fig. 1c, d). RANK expressions were confirmed by flow cytometry, western blotting and qPCR of positive control with osteoclasts and a negative control with RANK silenced BMSCs (Figure S2a-c). As BMSCs are enriched in PαS cells and calvarial cells are used as osteoblasts, we isolated these two cells and examined (Figure S3a). RANK was expressed in both cell types and decreased after osteogenic differentiation (Figure S3b). In vivo expression was determined by in situ immunostaining with CD90 and RANK in the secondary spongiosa of the distal femur (Fig. 1e). The results indicate that RANK is highly expressed in BMSCs.

### RANKL signaling inhibits osteogenic differentiation of BMSCs

**Fig. 2:**
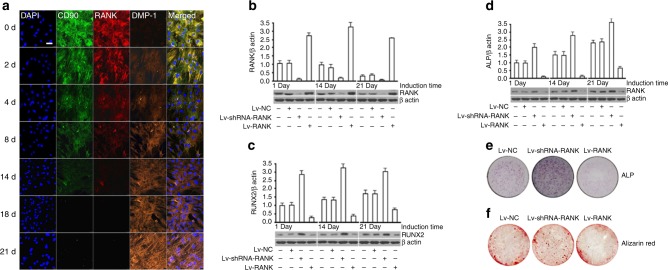
RANK inhibits osteoblastic differentiation of BMSCs in vitro. **a** Immunofluorescence confocal analysis of CD90, RANK, and DMP-1 after osteogenic induction of human BMSCs from 0 to 21 d. Scale bar = 50 μm. **b** Western blot analysis of RANK (66 kD) expressions after osteogenic induction with RANK overexpression and knockdown in human BMSCs at 0 d/14 d/21 d. **c** Western blot analysis of RUNX2 (56 kD) after osteogenic induction with RANK overexpression and knockdown in human BMSCs at 0 d/14 d/21 d. **d** Western blot analysis of ALP (39 kD) after osteogenic induction with RANK overexpression and knockdown in human BMSCs at 0 d/14 d/21 d. β-actin was used as an endogenous reference for normalization. **e** and **f** ALP and alizarin red staining after 21 days of osteogenic induction of human BMSCs

RANKL has been reported to be expressed in BMSCs.^17^ To determine the changes of RANK during osteogenesis and adipogenesis in vitro, we induced osteogenic differentiation of BMSCs and detected the expressions of RANK with triple immunofluorescence staining of CD90, RANK, and dentin matrix protein 1 (DMP-1), an osteoblast marker at different time points from 0 to 21 d. Results showed that after the osteoblastic differentiation began, the expressions of CD90 and RANK were rapidly downregulated, while the DMP-1 expression was gradually increased (Fig. 2a, b).

To test its function, we used RANKL to activate RANK in vitro and found that RANKL (50 ng·ml^−1^) significantly inhibited calcium nodules formation by alizarin red staining (Figure S4). Then we over-expressed and knocked-down RANK in BMSCs (Figure S5) and induced osteogenic and adipogenic differentiation. Western blot analysis showed that osteoblastic differentiation was significantly enhanced after *rank* was silenced demonstrated by significantly increased alkaline phosphatase (ALP) and runt-related transcription factor 2 (RUNX2) expressions, while RANK overexpression significantly inhibited osteoblastic differentiation (Fig. 2c, d). ALP and alizarin red staining results showed the osteoblastic differentiation of BMSCs was consistent with western blotting (Fig. 2e, f).

Interestingly, RANK did not affect adipogenic differentiation of BMSCs. After the adipogenic differentiation was induced, CD90 rapidly decreased while RANK expression did not change significantly (Figure S6a). Western blot showed that overexpression or knockdown did not affect adipogenic differentiation by lipoprotein lipase (LPL) and peroxisome proliferator-activated receptor γ2 (PPARγ2), two adipogenesis markers (Figure S6c-d) and oil red O staining (Figure S6e). The results suggest a crucial role of RANK in regulating osteoblastic differentiation of BMSCs at an early stage.

### Knockout of rank in BMSCs increases bone formation in mice

As whole-body loss-of-function approach will inevitably affect osteoclastogenesis, we used a cell-specific gene knockout approach. We crossed floxed *rank* mice with *Prx1*-*Cre* mice to generate MSCs conditional *rank* knockout mice (named *rank*^−/−^) (Figure S7a-b). *Rank*^flox/flox^ control littermates were referred to as *rank*^+/+^. We compared sex-matched littermates using paired statistical tests to evaluate difference significance of independent experiments. Both male and female mice were used in the study and all the conclusions were based on analysis of both male and female mice. But only female data were presented. The body weight and length of *rank*^−/−^ and *rank*^+/+^ mice were similar at birth, but they increased faster in *rank*^−/−^ mice for both male and female (Figure S7c). No difference was detected for bone analysis between *rank*^+/+^ and WT mice.

**Fig. 3:**
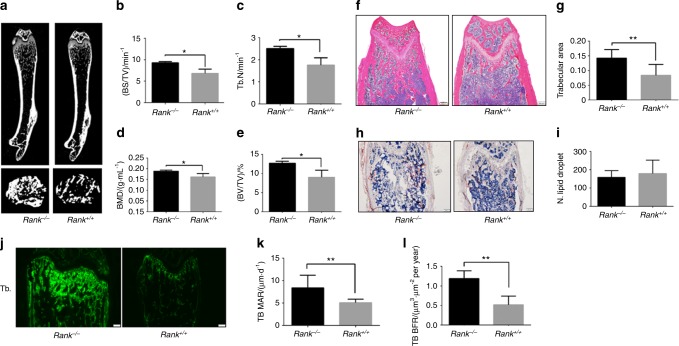
Increased bone formation during bone remodeling in *rank*^*−/−*^ mice. **a** Representative micro-CT images of distal femurs from 8-week-female adult *rank*^+/+^ and *rank*^−/−^ mice. **b**–**e** Quantitative micro-CT analysis of the secondary spongiosa of distal femora. **b** Bone surface density (BS/TV), **c** Trabecular number (Tb.N), **d** Volumetric BMD, **e** Bone volume fraction. **f** H&E staining of the distal femora. Scale bar = 200 μm. **g** Trabecular area analysis. **h** Oil Red O staining of the distal femora. Scale bar = 200 μm. **i** Lipid droplet analysis. **j** Calcein double labeling of the metaphyseal trabecular bone (scale bar = 200 μm). **k** Trabecular mineral apposition rate analysis. **l** Trabecular bone (TB) formation rate. All data are mean ± s.e.m. *n* = 8. *P < 0.05, **P < 0.01 by one-way analysis of variance (ANOVA) followed by *t*-test

BMD was greater in 8-week-old *rank*^−/−^ mice than in their *rank*^+/+^ littermates of the same age and gender. *Rank*^−/−^ mice had a significantly higher trabecular bone volume, trabecular number, and bone surface in distal femur but not in the spine relative to their *rank*^+/+^ littermates (*P* < 0.05) (Fig. 3a–e, Figure S8a, c). However, the cortical bone mineral density, porosity, and thickness were not statistically different in two groups (Figure S8b). Similar results were found in H&E-stained sections and in histomorphometric analyses (Fig. 3f, g). Oil red O staining showed that the number of fat vacuoles in *rank*^−/−^ mice was not significantly different from that in their *rank*^+/+^ littermates (Fig. 3h, i). Calcein double labeling results showed a significantly higher bone formation rate (BFR) and mineral apposition rate (MAR) of trabecular bone in *rank*^−/−^ mice than in *rank*^+/+^ mice (Fig. 3j, l). The above results indicate RANK ablation promoted trabecular bone formation in adult mice.

### Osteoblasts increase in rank^−/−^ mice

**Fig. 4:**
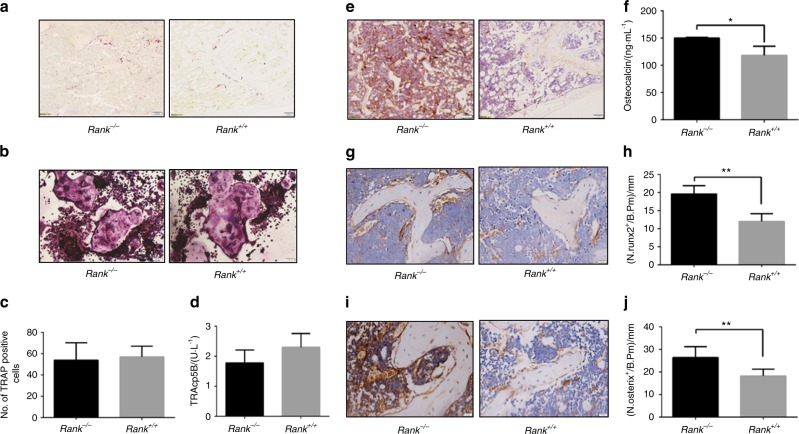
Increased osteoblast maturation in *rank*^*−/−*^ mice. **a** Light micrographs of TRAP staining performed on trabecular bone sections from distal femora. Scale bar = 100 μm. **b** Formation of TRAP-positive cells from bone mononuclear cells isolated from *rank*^−/−^ and *rank*^+/+^ mice. Scale bar = 100 μm. **c** Quantification of mature osteoclasts. **d** Serum TRAcp5b levels of 8-week-female adult *rank*^*+/+*^ and *rank*^*−/−*^ mice. **e** Osteocalcin immunohistological staining of the metaphyseal trabecular bone at distal femora. Scale bar = 100 μm. **f** Serum OCN levels of 8-week-female adult *rank*^*+/+*^ and *rank*^*−/−*^ mice. **g**–**h** Immunohistochemical analysis of runx2 in the metaphyseal trabecular bone at distal femora. Scale bar = 20 μm. **i**–**j** Immunohistochemical analysis of osterix in the metaphyseal trabecular bone at distal femora. Scale bar = 20 μm. All data are mean ± s.e.m. *n* = 8. *P < 0.05, **P < 0.01 by one-way ANOVA followed by *t*-test

To determine whether the increased BMD in the *rank*^−/−^ mice was the result of decreased osteoclasts, we measured the number of mature osteoclasts by tartrate-resistant acid phosphatase (TRAP) staining and serum TRAcp5b, CTX-1 levels. The number of TRAP^+^ mature osteoclasts in *rank*^−/−^ mice was not significantly different from that in *rank*^+/+^ littermates (Fig. 4a). Formation of TRAP-positive cells from bone marrow mononuclear cells from *rank*^−/−^ and *rank*^+/+^ mice and osteoclasts quantification showed no significant difference (Fig. 4b, c). The serum level of TRAcp5b in *rank*^−/−^ mice was slightly lower than that in *rank*^+/+^ mice, but the difference was not statistically significant (Fig. 4d). Similar results were found for CTX-1 (data not shown). Moreover, the tooth eruption of *rank*^−/−^ mice was normal (data not shown). The results demonstrate that conditional knockout of RANK in BMSCs has little effects on osteoclastogenesis.

Then we measured the number of mature osteoblasts by immunostaining femur sections. The number of osteocalcin (OCN) positive mature osteoblasts on the bone surfaces and the serum OCN levels of *rank*^−/−^ mice were significantly higher than that in *rank*^+/+^ littermates (Fig. 4e, f). We then measured the numbers of osteoblasts at different stages of differentiation by immunostaining femur sections. The numbers of runx2^+^ (Fig. 4g, h) and osterix^+^ osteoprogenitors (Fig. 4i, j) on the trabecula of rank^−/−^ mice were significantly higher than those in their *rank*^+/+^ littermates.

As the *Prx1*-*Cre* transgene causes deletion in both chondrocytes and osteoblast-lineage cells, to exclude the interference of chondrocytes, we isolated chondrocytes and performed the immunohistological staining of RANK in vitro and in vivo on the distal femur sections from *rank*^+/+^ and *rank*^−/−^ mice at 8-week. It showed that the chondrocyte did not express RANK (Figure S9a-c). The results show that RANKL signaling negatively regulates osteoblast formation in mice.

### RANKL signaling increases degradation and inhibits synthesis of β-catenin

**Fig. 5:**
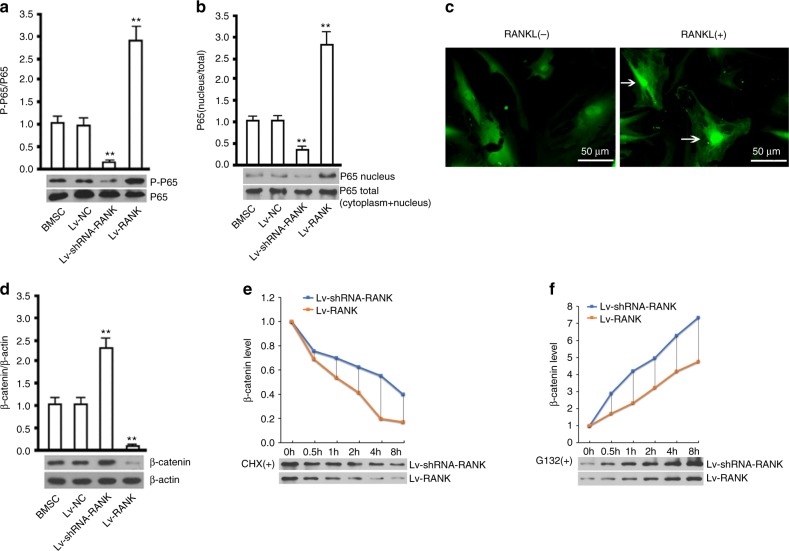
RANK affects β-catenin synthesis and degradation. **a** Western blot analysis of p-p65 versus p65 (65 kD) with RANK over-expressed and silenced in BMSCs from mice. **b** Western blot analysis of p65 (65 kDa) content in nucleus versus total of BMSCs. **c** Immunofluorescence images of p65 in BMSCs with sRANKL introduction (10 ng·mL^−1^), Scale bar = 50 μm. **d** Western blot analysis of β-catenin (98 kD) with RANK overexpressed and silenced in BMSCs. **e** Analysis of β-catenin degradation rate with RANK overexpressed and silenced. **f** Analysis of β-catenin synthesis with RANK overexpressed and silenced. All data are the mean ± s.e.m. of triplicate cultures. **P < 0.01 by one-way ANOVA followed by *t*-test

To investigate the possible underlying mechanisms, we explored the effects of RANKL signaling on β-catenin, a key transcriptor for BMSCs osteoblastic differentiation. Overexpression of RANK significantly increased phosphorylation which was decreased by RANK knockdown (Fig. 5a). Exogenous RANKL (10 ng·mL^−1^) added increased p65 nucleus translocation (Fig. 5b, c). RANK and β-catenin expressions were reversely correlated. Overexpression of RANK significantly reduced β-catenin content (Fig. 5d). CHX stands for cycloheximide and is used to inhibit protein synthesis. G132 is MG132 (Carbobenzoxy-L-leucyl-L-leucyl-L-leucinal), a proteasome inhibitor used to inhibit protein ubiquitylation and degradation. RANK overexpression increased β-catenin degradation and inhibited its synthesis (Figs. 5e, f and 7a).

To further illustrate the relationship between p65 and β-catenin, we evaluated the effects of p65 and β-catenin silence on the osteogenic differentiation of BMSCs. Western blotting results showed that p65 knockdown significantly promoted osteogenic differentiation demonstrated by higher runx2 and ALP expressions compared with induction group, which was consistent with the previous report,^18^ while knockdown of β-catenin significantly abolished the effects. Silencing both p65 and β-catenin significantly inhibited osteogenesis. The results implied that p65 inhibited BMSCs osteogenic differentiation dependent on β-catenin (Figure S10). RANKL signaling inhibits osteoblastic differentiation of BMSCs through inhibiting β-catenin synthesis and promoting its degradation.

Since BMSCs express RANKL, the ligand for RANK, we explored the roles of RANKL in BMSCs osteogenic differentiation. Similar to RANK, RANKL overexpression significantly inhibited BMSCs osteogenic differentiation while RANKL silencing promoted BMSCs osteogenic differentiation demonstrated by ALP and alizarin red stainings (Figure S11a-b). RANKL overexpression significantly activated phosphorylation of p65 and promoted nucleus translocation of p65 while silencing inhibited p65 activation (Figure S11c-d). RANKL overexpression significantly decreased β-catenin while silencing increased β-catenin (Figure S11e). Interestingly, RANKL expression is consistent with RANK (Figure S11f).

### RANK knockout BMSCs ameliorates ovariectomy-induced bone loss

**Fig. 6:**
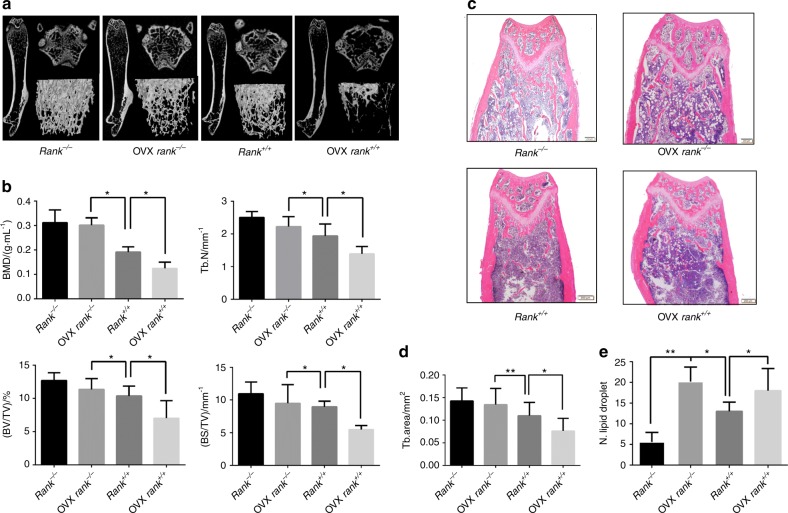
RANK^−/−^ in BMSCs resists OVX-induced pathological bone loss. **a** Representative micro-CT images of distal femurs from 14-week-female adult *rank*^+/+^ and *rank*^−/−^ mice after 6 weeks of ovariectomy. **b** Analysis of bone mineral density (BMD), bone surface density (BS/TV), trabecular number (Tb.N), bone volume over total volume (BV/TV). **c** HE staining of the distal femora. Scale bar = 200 μm. **d** Trabecular area analysis. **e** Analysis of fat vacuole number. All data are mean ± s.e.m. *n* = 8. *P < 0.05, **P < 0.01 by one-way analysis of variance (ANOVA) followed by *t*-test

To explore the effects of RANK knockout in BMSCs on pathological bone loss, we used the OVX-induced bone loss model. After 6 weeks of ovariectomy, *rank*^*+/+*^ mice showed a significant bone loss by HE staining and micro-CT analysis of the distal femora (Fig. 6a). *Rank*^*−/−*^ mice showed significant resistance to OVX-induced bone loss (Fig. 6a). BMD, Tb.N, BV/TV and BS/TV were greater in *rank*^−/−^ ovariectomized mice than in their ovariectomized *rank*^+/+^ littermates and similar to their sham *rank*^−/−^ mice (*P* *>* 0.05) (Fig. 6b). But *rank*^−/−^ mice had a significant decrease in BMD, Tb.N, BV/TV and BS/TV in the vertebrae (*P* < 0.05) (Figure S12). Similar results were found in HE-stained sections and in histomorphometric analyses (Fig. 6c). The number of fat vacuoles in *rank*^−/−^ ovariectomized mice was not statistically different from that of the *rank*^+/+^ ovariectomized mice (Fig. 6e). The ameliorated bone loss induced by OVX is attributed to the increased bone formation.

**Fig. 7:**
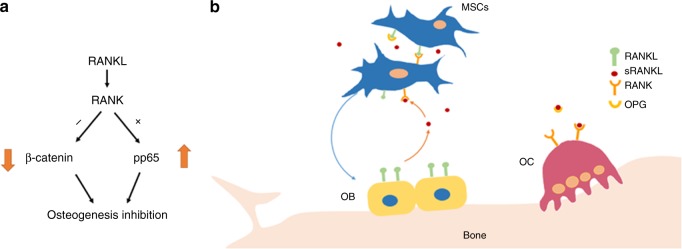
Illustrations of BMSCs osteoblastic differentiation regulations. **a** Molecular mechanisms of RANK in regulating the osteogenic differentiation of BMSCs. **b** Schematic diagram RANKL signaling regulating the osteogenic differentiation of BMSCs. The RANKL-RANK interaction between BMSCs restrains osteoblastic differentiation. Soluble RANKL released from osteoblasts negatively regulates osteoblastic differentiation of BMSCs

## Discussion

RANKL signaling is essential for osteoclast development and RANK^−/−^ mice showed profound osteopetrosis due to a lack of mature osteoclasts.^7,8^ RANK^−/−^ mice were characterized by small body size, shortened limbs and doming of the skull. For decades studies of RANK have been focusing on osteoclastogenesis.^19,20^ And RANKL inhibitor denosumab has been used clinically to inhibit osteoclastogenesis as an anti-resorptive agent.^9^

BMSCs are progenitor cells of osteoblasts in bone marrow. RANK expression in BMSCs has not been determined. In 2015, a study showed that RANK was expressed in osteosarcoma tumor cells and associated with osteoblast chemotactic migration.^16^ Based on the above findings, we hypothesized that RANKL signaling could play an important role in bone formation regulation.

In this study, we first isolated human and mice BMSCs and identified RANK expressions in vitro and in vivo. After the osteogenic differentiation began, the expression was rapidly downregulated. Intriguingly, RANK expression was not affected during adipogenic differentiation. Thus, we speculated that RANK functioned in regulating osteoblastic differentiation. RANKL signaling inhibited osteoblastic differentiation of BMSCs in vitro. To avoid interfering with osteoclastogenesis by general knockout, we used the conditional knockout technique to specifically knockout RANK in BMSCs. We found that RANK elimination in BMSCs significantly increased bone formation with more osteoblasts by BMSCs and adipogenesis by BMSCs was not affected. The osteoclasts seemed not being affected. Thus, the increased bone formation by loss of RANK in BMSCs is independent of osteoclastogenesis.

As the ligand for RANK receptor, RANKL has been reported to couple bone resorption and formation.^14^ Vesicular RANK secreted from the maturing osteoclasts binds osteoblastic RANKL and promotes bone formation by triggering RANKL reverse signaling. Schena et al. reported that RANKL-deficient BMSCs displayed an osteogenic differentiation defect in vitro and in vivo.^21^ We replicated the in vitro experiments and unexpectedly found that RANKL-deficient BMSCs showed increased osteogenic capacities and RANKL overexpression inhibited BMSCs osteogenic differentiation, which is controversial to reported study. The in vivo protocol employed is ectopic bone formation assay, which could not fully reflect the roles of RANKL-deficient BMSCs. For, an in vivo study by Jinhu Xiong et al. reported that mice with RANKL deletion in committed osteoblast progenitors with Osx1-Cre showed a normal cancellous architecture with no evidence of bone formation defect.^17^ Generally, the roles of RANKL on BMSCs in regulating osteoblastogenesis are under debate, which needs further study.

For molecular mechanisms, after RANKL activates RANK, several pathways, including NF-κB, MAPK and Akt are activated. NF-κB was reported to inhibit osteoblastic bone formation.^18^ We hypothesized that NF-κB pathway was vital. RANK activation or overexpression led to increased phosphorylation and translocation of p65 as well as decreased β-catenin expressions. β-catenin is a crucial component of the *Wnt* pathway, which is vital for osteoblastic differentiation.^22^ β-catenin and p65 could form a dynamic complex which could undergo temporal dissociation to allow for p65 activation and the decreased β-catenin is associated with increased p65 activity.^23^ The upstream kinase of p65, IKKβ is also a β-catenin kinase that phosphorylates the conserved degron motif to prime the β-catenin for β-transducing repeat-containing protein (βTrCP)-mediated ubiquitination and degradation in MSCs.^24^ Thereafter, adipogenesis is increased while the osteogenesis is inhibited. Therefore, how RANKL signaling regulates BMSCs osteoblastic differentiation is generally clear. Once the RANK is activated by RANKL, TRAF6 is recruited and IKKβ is activated and phosphorylates IκB and β-catenin. Then the complex dissociates with p65 released, phosphorylated and translocated into the nucleus and β-catenin phosphorylated and degraded through inducing βTrCP expression. The βTrCP targets both β-catenin and IκB to induce ubiquitination and subsequent degradation,^25^ forming a positive feedback for the NF-κB pathway sustained activation and Wnt/β-catenin inhibition.

In this study, we expanded the understanding for RANKL signaling and found that it not only promoted osteoclastogenesis but negatively regulated osteoblastic differentiation of BMSCs and postnatal bone formation in bone modeling and remodeling (Fig. 7b).

## Materials and methods

### BMSCs isolation and induction

Human BMSCs were isolated from three premenopausal female patients receiving fracture surgeries. The procedure was approved by the Ethics committee of Shanghai Changhai hospital and the informed consent was obtained from all subjects. Bone marrow cells were flushed from the cavities of femurs and tibias of 8-week-old *rank*^−/−^ mice and *rank*^+/+^ control littermates and were plated in α-MEM with 15% FBS (Fetal bovine serum) and 1% penicillin/streptomycin (Invitrogen) at 37 °C in a 5% CO_2_ incubator. After 48 h of culture, the medium was changed to remove non-adherent cells. Then cells were cultured for an additional 4 days with a single medium change until BMSCs reached confluency. Cells were passaged with 0.25% trypsin-EDTA digestion (Invitrogen) and reseeded at 5 × 10^5^ cells/6-well or 1 × 10^5^ cells/12-well. Third-passage BMSCs were subjected to induction of adipogenic and osteogenic differentiation, and transfection of plasmids. For osteogenic induction, BMSCs were cultured in media (α-MEM containing 15% FBS, 1% penicillin/streptomycin, 50 μg·mL^−1^ ascorbic acid and 10 mmol·L^−1^ β-glycerophosphate) for 21 days. Alizarin Red staining was carried out to determine the mineralization. For adipogenic differentiation assay, BMSCs were cultured in 6-well plates with adipogenesis induction medium (α-MEM, 10% FCS, 0.5 mmol·L^−1^ 3-isobutyl-1-methylxanthine, 5 μg·mL^−1^ insulin, and 1 μmol·L^−1^ dexamethasone) for 14 days. Oil Red O staining was performed to detect mature adipocytes.

### Animal experiments

To generate conditional *rank* knockout mouse models for investigating postnatal bone formation and remodeling, we first crossed female *rank*^flox/flox^ mice (B6.Cg-Tnfrsf11atm1.1Irw/J) with male *Prx1*-*Cre* mice (B6.Cg-Tg(*Prx1*-*Cre*)1Cjt/J) from the Jackson laboratory and obtained *Prx1-Cre*: *rank*^flox/‒^ mice among which male mice were crossed with *rank*^flox/flox^ mice to obtain *Prx1-Cre*: *rank*^flox/flox^ mice for study. All skeletal analyses were performed on both female and male mice unless otherwise specified. Data of female mice were provided. For serum TRAcp5B, CTX-1 and OCN assay, serum was collected from 8-week-old mice without fasting following the manufacturer’s instructions.

### Histology and TRAP staining

For histological analysis, decalcified distal femur and vertebrae samples were embedded in paraffin and then sectioned at 4-μm thickness. TRAP, OCN staining was performed following standard protocols.

### Bone structure analysis

The bone structure of the distal femur and vertebra was analyzed using micro-computed tomography (Micro CT) (Skyscan1172, Antwerp, Belgium). The analysis parameters were 80 kv, 124 μA and resolution was 8 μm. Structural parameters of metaphyseal area and trabecular bone were analyzed with the built-in software. Total bone mineral density (BMD), bone volume/total volume (BV/TV), trabecular number (Tb.N), bone surface area expressed per unit total volume (BS/TV) were analyzed. Two-dimensional and three-dimensional bone structure image slices were reconstructed.

### RT-PCR

Total RNA was extracted following the instruction of TRIzol Reagent (Invitrogen). RNA (2 µg) was used to synthesize cDNA by reverse transcription Master Mix (Promega). Quantitative real-time PCR was performed with iTaq Universal SYBR Green Supermix (Bio-Rad) on a CFX384 Real-Time PCR system (Bio-Rad). Data were normalized by 18S rRNA transcript and calculation was performed with the ∆∆Ct method.

Rank-RT(human)-F: CTGTCTTCAGGAGATTGGCA, rank-RT(human)-R: ACTTGGAAATCTG-GCAGCTC; RANK(mouse)-RT-F: ATCCAGCAGGGAAGCAAA, RANK(mouse)-RT-R: GGGACACGGGCATAGAGT.

### Western blotting

Protein quantification was performed. Protein lysate was introduced to SDS-PAGE and subsequently electrotransferred to a polyvinylidene fluoride membrane. Western blotting was carried out as previously reported.^26^ The antibodies used were listed as follows: anti-RANK (Abcam, ab200369, 1:800), anti-RUNX2 (Cell Signaling Technology, 12486, 1:1 000), anti-ALP (Millipore, 06-942, 1:1 000), anti-pp65 (Abcam, ab76307, 1:5 000), anti-p65 (Active Motif, 39159, 1:1 000), anti-β catenin (Cell Signaling Technology, 4824, 1:200).

### Silence and overexpression of RANK and RANKL in mouse BMSCs by lentivirus

A siRNA sequence complementarily binding to mouse RANK (NM_017206.1) was chosen. The target sequences of siRNA (5′-GGTTGTCTACTTCACTGCT-3′) are homologous to RANK, respectively. The target sequences of siRNA (5′-GCGTACCTACAGACTATCT-3′) are homologous to RANKL. The oligonucleotide templates of these shRNAs were chemically synthesized and cloned into the linear pSIH1-H1-copGFP shRNA Vector (System Biosciences) which was obtained through digestion by BamH I and EcoR I (Takara, Dalian, China) and purification by agarose gel electrophoresis. An invalid siRNA sequence (5′-GTTGTCATGTCTATCTCGC-3′) was used as an NC (negative control). The CDS (Coding sequence) of mouse RANK was amplified using the primers 5′-GGAATTCGCCACCATGGCCACCAAGGAGAAG-3′ and 5′-CGGGATCCTCACATCATGGTCTCCAC-3′, which contain an EcoRI cutting site and Kozak sequence and a BamHI cutting site, respectively. The cDNA was prepared by reverse transcription RNA isolated from rat brain tissue. The PCR product was digested and cloned into a pcDH1-CMV lentiviral expression vector (System Biosciences); the recombinant vector was named pcDH1- RANK. The products of the vectors were confirmed by DNA sequencing, and endotoxin-free DNA was prepared.

Packaging of lentivirus was carried out in 293 virus packaging cell line. One day before transfection, 293TN cells were seeded into 10-cm dishes. Two micrograms of each shRNA and expression vector and 10 μg pPACK Packaging Plasmid Mix (System Biosciences) was co-transfected using Lipofectamine 2000 (Invitrogen) in accordance with the manufacturer’s protocol. The medium was replaced with DMEM plus 1% FBS. Forty-eight hours later, the supernatant was collected and then cleared by centrifugation at 5 000*×g* at 4 °C for 5 min, and passed through a 0.45 µm PVDF membrane (Millipore, MI, USA). The titer of the virus was determined by gradient dilution. The packaged lentiviruses were named as Lv-shRNA-RANK, Lv-NC, and Lv-RANK.

P3 mouse BMSCs in logarithmic phase were seeded on 6-well plates at 5 × 10^5^ cells/well. One day later, the viral solution was added at an MOI of 20. The infection efficiency was evaluated by observing and analyzing the fluorescent mark 72 h after infection. And total RNA or total protein were isolated from the cells and subjected to real-time PCR for RANK mRNA or protein level, respectively.

### Immunofluorescence and immunohistological assay

hMSCs and mMSCs were fixed with formaldehyde (4% in PBS), permeabilized with Triton X-100 (0.4% in PBS), incubated with blocking buffer (10% donkey serum in PBS), and stained with primary antibody overnight at 4 °C. Then, cells were treated with secondary antibodies for 1 h at room temperature. Hoechst 33342 (Invitrogen) was used for nuclear DNA staining. The antibodies used are as follows: anti-RANK (ab12008, 1:100), anti-CD90 (Abcam, ab21624, 1:200), anti-DMP1 (Santa Cruz, sc-17320, 1:100).

### Flow cytometry

The procedure was carried out according to the protocals. The antibodies used are as follows: anti-RANK (ab13918, 1:50), anti-Runx2 (ab192256, 1:150), anti-ALP (ab197781, 1:50), anti-CD73 (AF4488, 1:200), anti-CD90 (ab3105, 1:100), anti-CD105 (ab221675, 1:100), anti-CD34 (ab8158, 1:100), anti-CD45 (ab10558, 1:100), anti-PDGFRα (ab203491), anti-SCA1 (ab93537).

### Statistical analysis

The data were expressed as means ± SD. The two independent-sample *t*-test was used for comparisons between two groups. In cases of a comparison involving more than 2 groups, a one-way ANOVA was used. Statistical significance was considered as *P* < 0.05.

## Electronic supplementary material

Responses

supplemental materials

## Data Availability

The authors declare that all data supporting the findings of this study are available within the paper and its supplementary information files.
