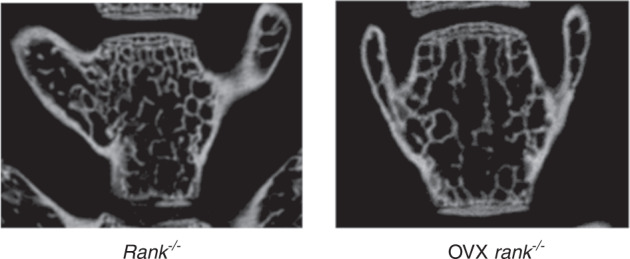# Correction to: RANKL signaling in bone marrow mesenchymal stem cells negatively regulates osteoblastic bone formation

**DOI:** 10.1038/s41413-022-00230-z

**Published:** 2022-08-15

**Authors:** Xiao Chen, Xin Zhi, Jun Wang, Jiacan Su

**Affiliations:** 1 Department of Orthopedics Trauma, Shanghai Changhai Hospital, Second Military Medical University, Shanghai, China; 2 Department of Chemistry, Fudan University, Shanghai, China; 3 School of Basic Medical Sciences, Second Military Medical University, Shanghai, China; 4 College of Life Science, Fudan University, Shanghai, China

Correction to: *Bone Research* 10.1038/s41413-018-0035-6, published online 27 November 2018

During a reread of our article previously published in Bone Research^1^, we regrettably found that the left representative image of the femur from the Rank^-/-^ group in figure 6a was mistakenly pasted during images assembly. Similarly, the representative figure S12a rank^-/-^ was misused during graphs assembly. We noticed that the figure 2f had very low resolution and we replaced it with the images with high resolution. The correct images have been replaced. The current figure S5a legend is incorrect. From the lab record, we found that in figure S5a, 1 and 2 showed fluorescence of BMSCs infected with lentivirus-NC after 60 h while 3 and 4 showed BMSCs infected with lentivirus-NC after 72 h to examine the infection rate.

The current Figure legend for Figure S5 is:

Figure S5. RANK overexpression and silence. (a) Left: fluorescence of BMSCs infected with lentivirus after 72 h. (1 and 2) was infected with Lv-RANK and (3 and 4) was infected with Lv-shRNA-RANK. Right: Detection of intracellular RANK protein expression, MOI = 20. Scale bar = 100 μm. (b) PCR quantification analysis of rank expressions. (c) Western blot analysis of RANK expressions. Data represent the mean ± SEM (*n* = 8). *P < 0.05, **P < 0.01.

The correct Figure legend for Figure S5 should be:

Figure S5. RANK overexpression and silence. (a) fluorescence of BMSCs infected with lentivirus. 1 and 2 showed fluorescence of BMSCs infected with lentivirus-NC after 60 h while 3 and 4 showed BMSCs infected with lentivirus-NC after 72 h to examine the infection rate. MOI = 20. Scale bar = 100 μm. (b) PCR quantification analysis of rank expressions. (c) Western blot analysis of RANK expressions. Data represent the mean ± SEM (*n* = 8). *P < 0.05, **P < 0.01.

We sincerely apologize for this oversight, but it does not affect any of the original conclusions.

Original figure 2f
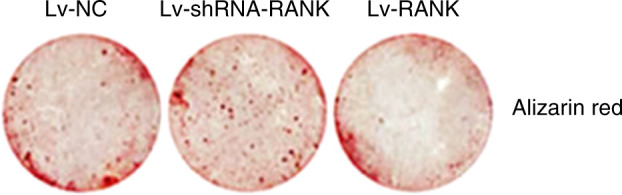


Corrected figure 2f
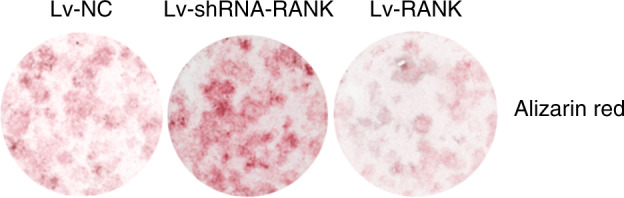


Original figure 6a
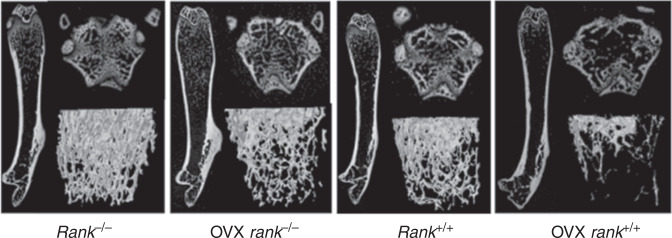


Corrected figure 6a
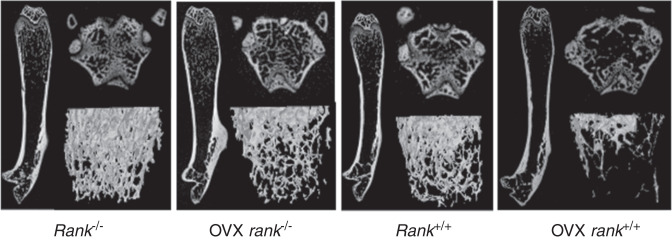


Original figure S12a
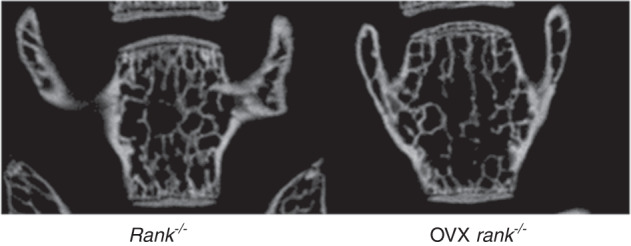


Corrected figure S12a