# Supplementary material for: RANKL signaling in bone marrow mesenchymal stem cells negatively regulates osteoblastic bone formation
Source: Bone Res. 2018 Nov 27;6:34. doi: 10.1038/s41413-018-0035-6 (PMC6255918; doi:10.1038/s41413-018-0035-6)
Supplement: Supplementary file 2 — supplemental materials [file 41413_2018_35_MOESM2_ESM.docx]

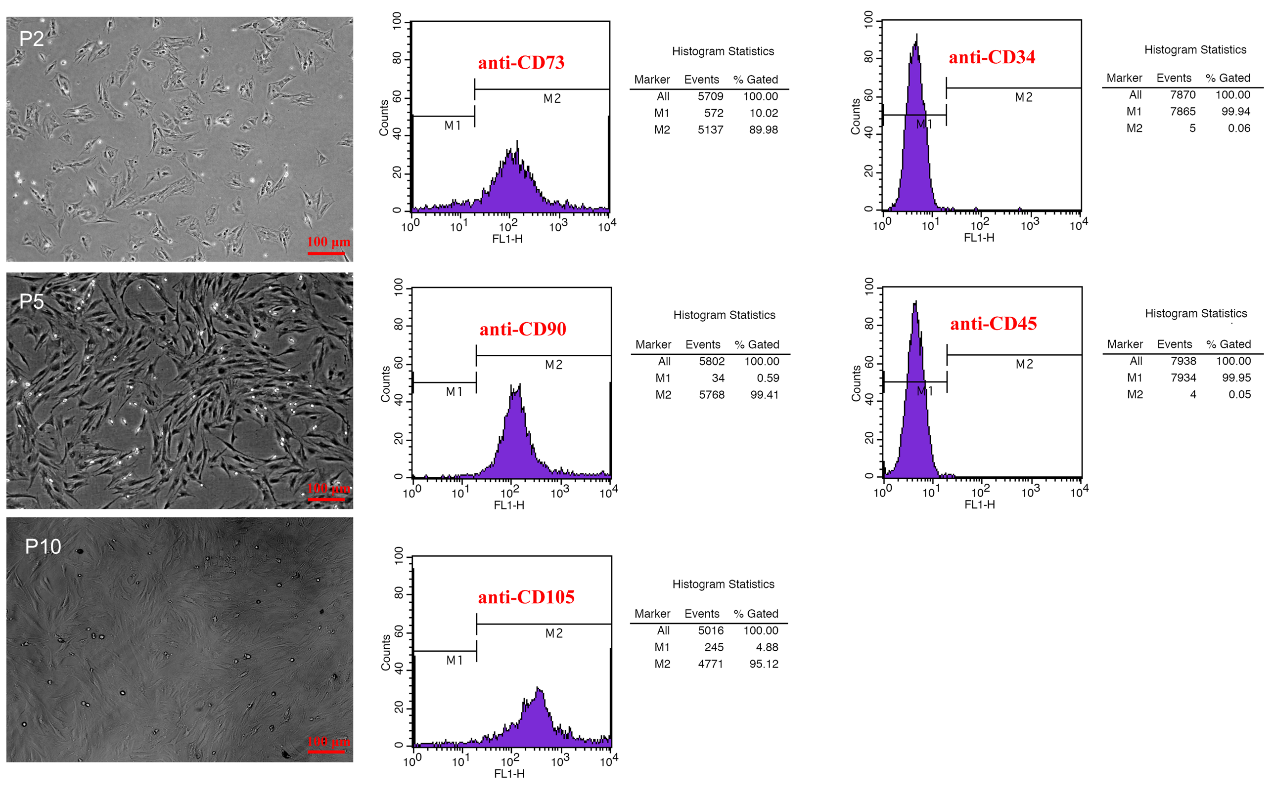


Figure S1. Identification of BMSCs from mice with flow cytometry

Mice BMSCs were identified with flow cytometry based on CD34^-^/CD45^-^/CD73^+^/CD90^+^/CD105^+^.


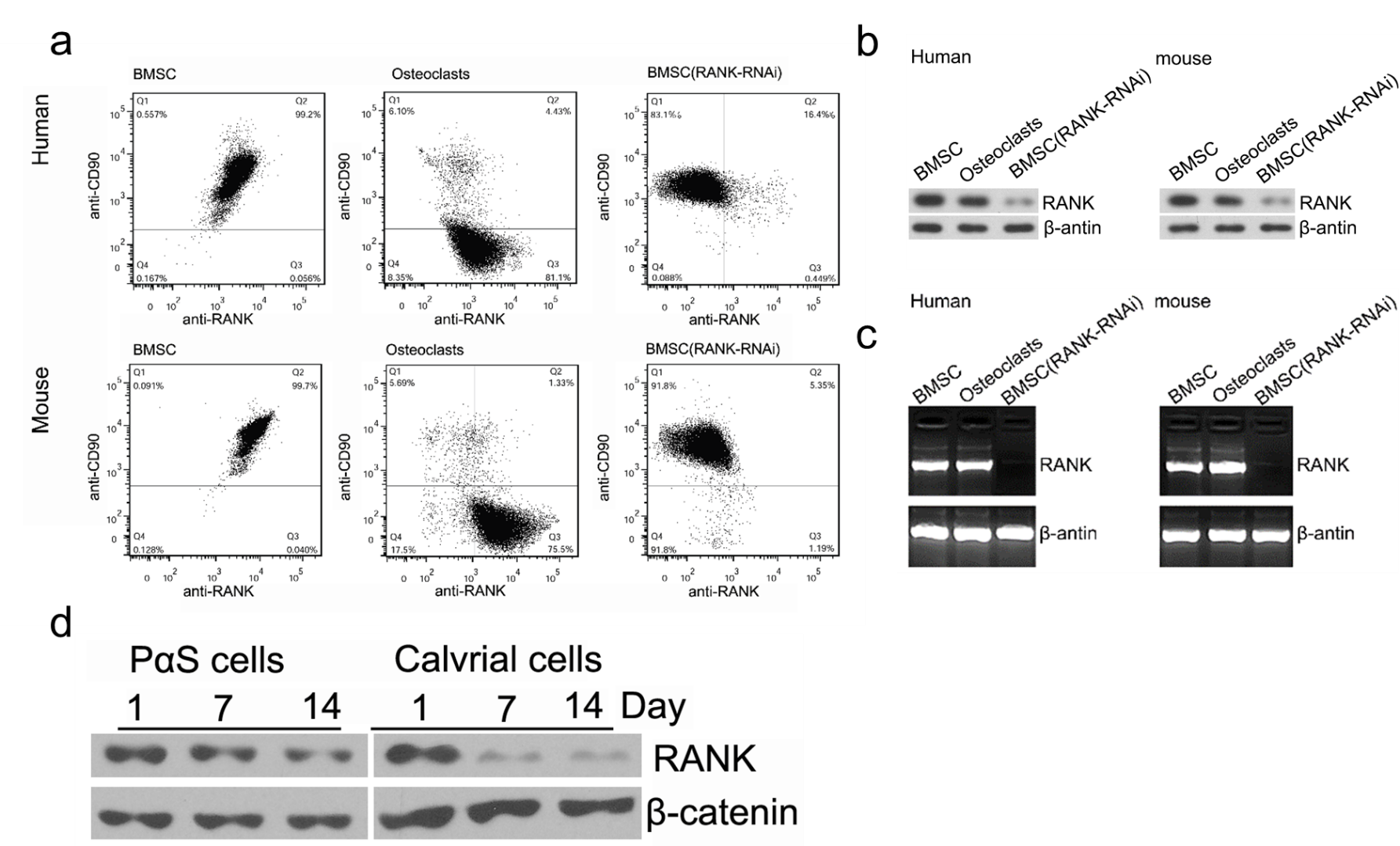


Figure S2. RANK expression in BMSCs. a-c. Flow cytometry, western blotting and PCR of RANK in BMSCs, osteoclasts and RANK silenced BMSCs.


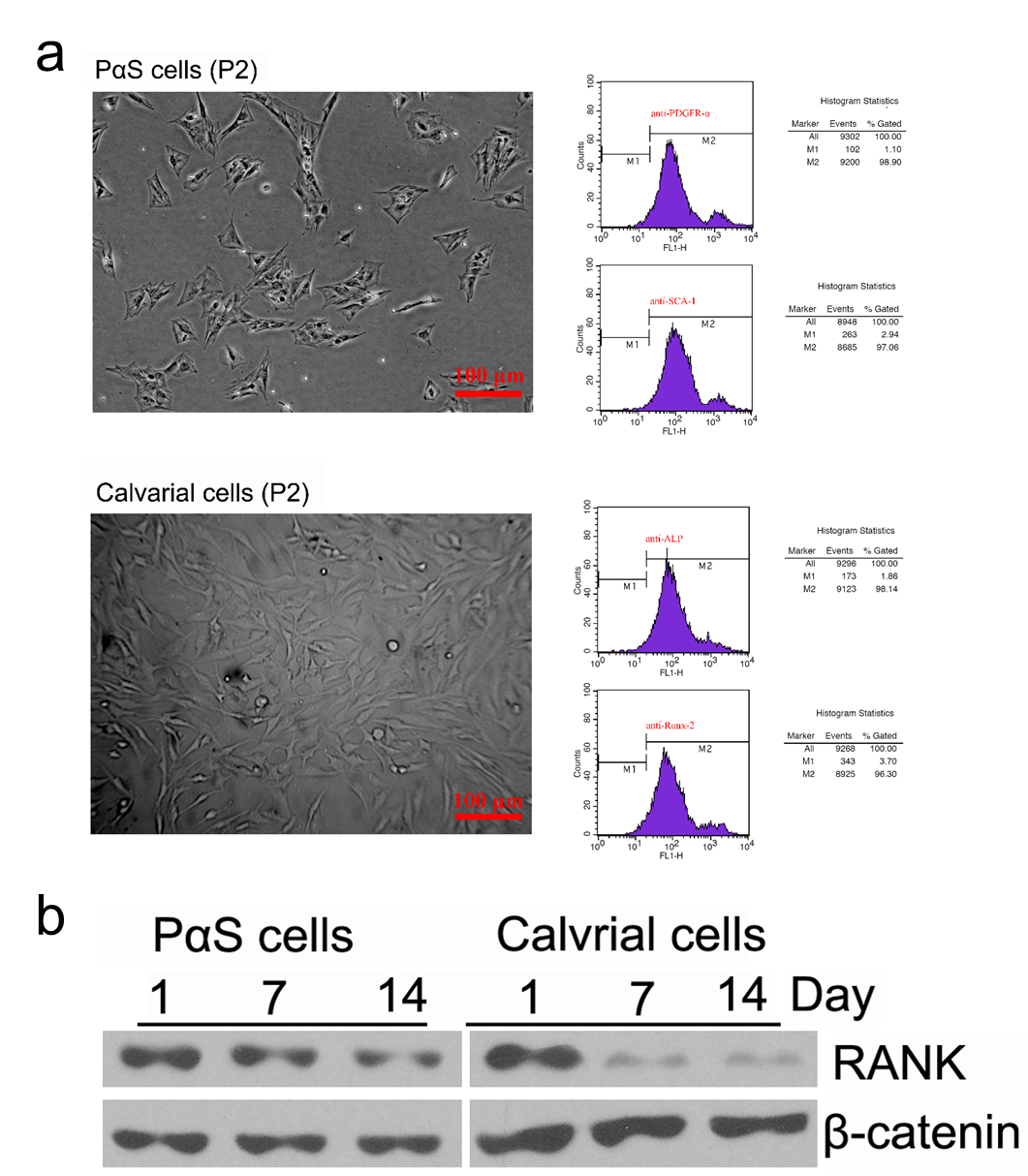


Figure S3. PαS cells and calvarial cells identification and RANK expressions. a. PαS cells were identified by PDGFR-α^+^/SCA-1^+^, while calvarial cells were identified by ALP^+^/Runx2^+^ with flow cytometry. Scale bar=100 μm. b. Western blotting of RANK in PαS cells and calvarial cells after osteogenic induction.


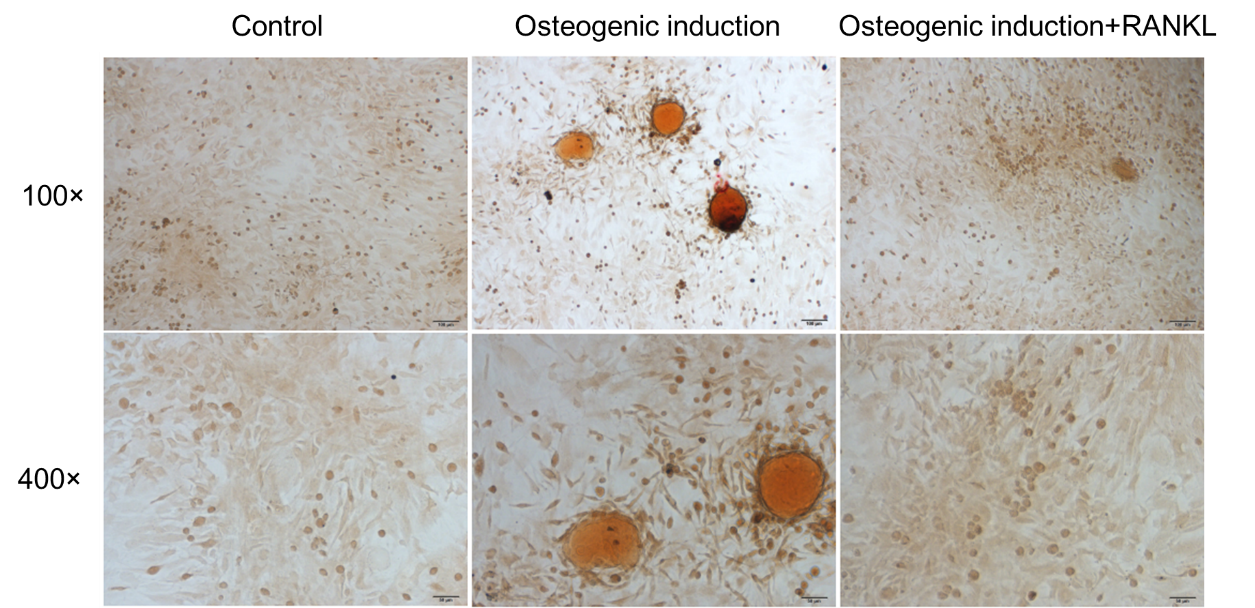


Figure S4. RANKL inhibits osteogenic differentiation of BMSCs. Alizarin Red S staining of calcium nodules after 21 days of osteogenic induction with and without RANKL.


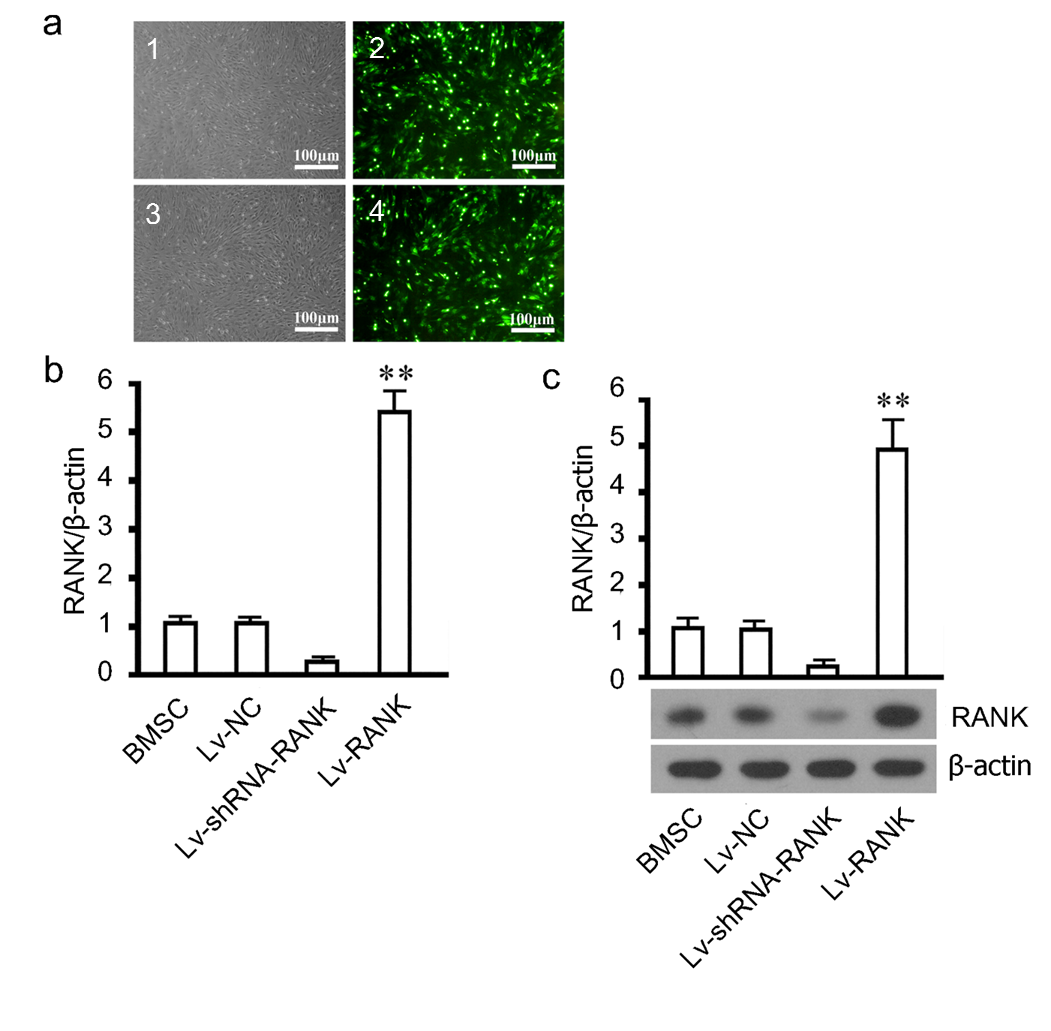


Figure S5. RANK overexpression and silence. (a) Left: fluorescence of BMSCs infected with lentivirus after 72 h. (1 and 2) was infected with Lv-RANK and (3 and 4) was infected with Lv-shRNA-RANK. Right: Detection of intracellular RANK protein expression, MOI=20. Scale bar=100 μm. (b) PCR quantification analysis of *rank* expressions. (c) Western blot analysis of RANK expressions. Data represent the mean ± SEM (n=8). **P*<0.05, ***P*<0.01.


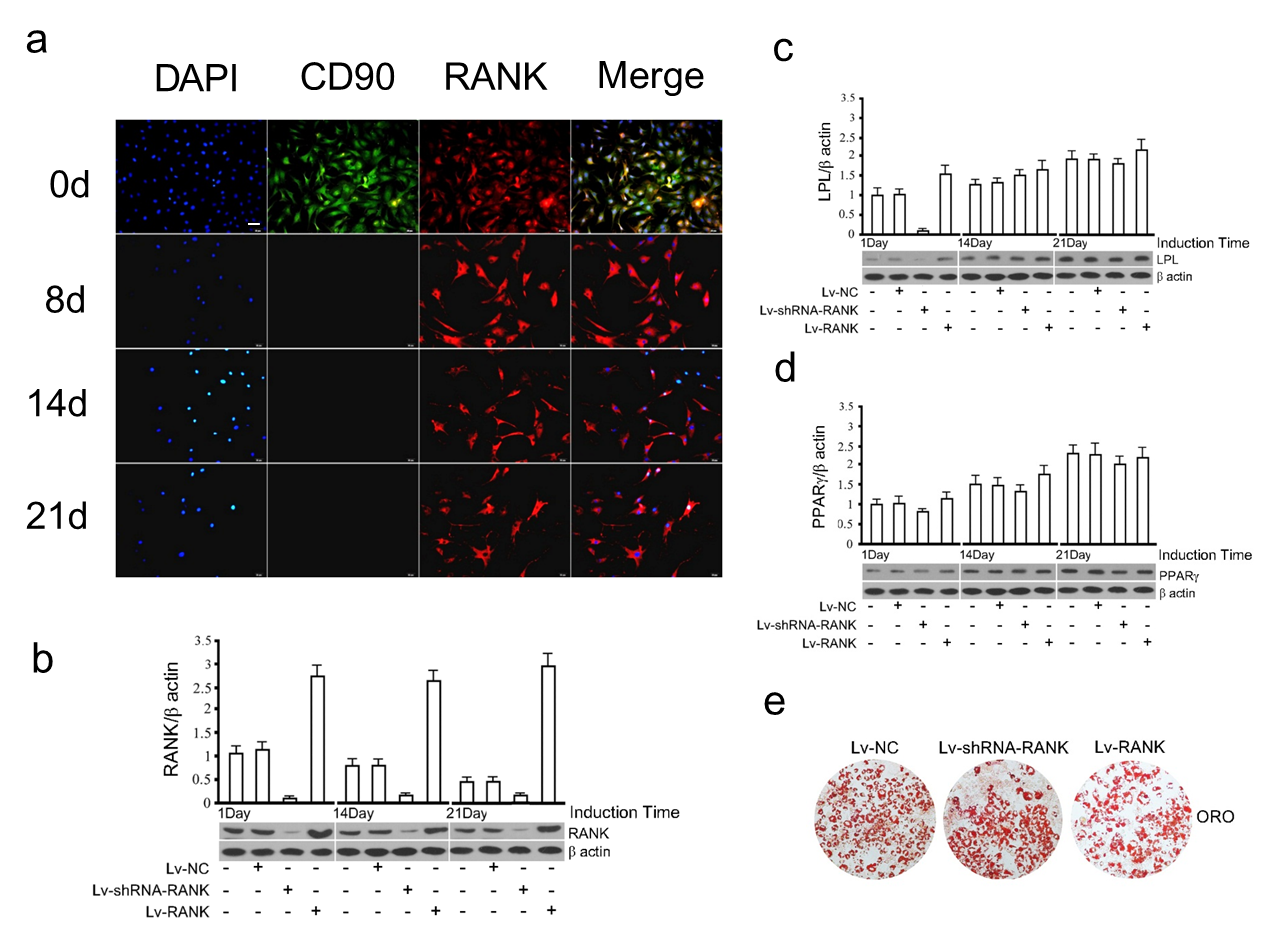


Figure S6. RANKL signaling does not affect adipogenic differentiation of BMSCs. (a) Immunofluorescence confocal analysis of CD90, RANK and DMP-1 after adipogenic induction of human BMSCs. Scale bar=100 μm. (b) Western blot analysis of RANK expressions after adipogenic induction with RANK overexpression and knockdown in mice BMSCs. (c) Western blot analysis of LPL after adipogenic induction with RANK overexpression and knockdown in mice BMSCs. (d) Western blot analysis of PPARγ2 after adipogenic induction with RANK over-expression and knockdown in mice BMSCs. (e) Oil O staining after 21 days of adipogenic induction of mice BMSCs with RANK overexpression and knockdown. All data are the mean + s.e.m. of triplicate cultures of BMSCs.


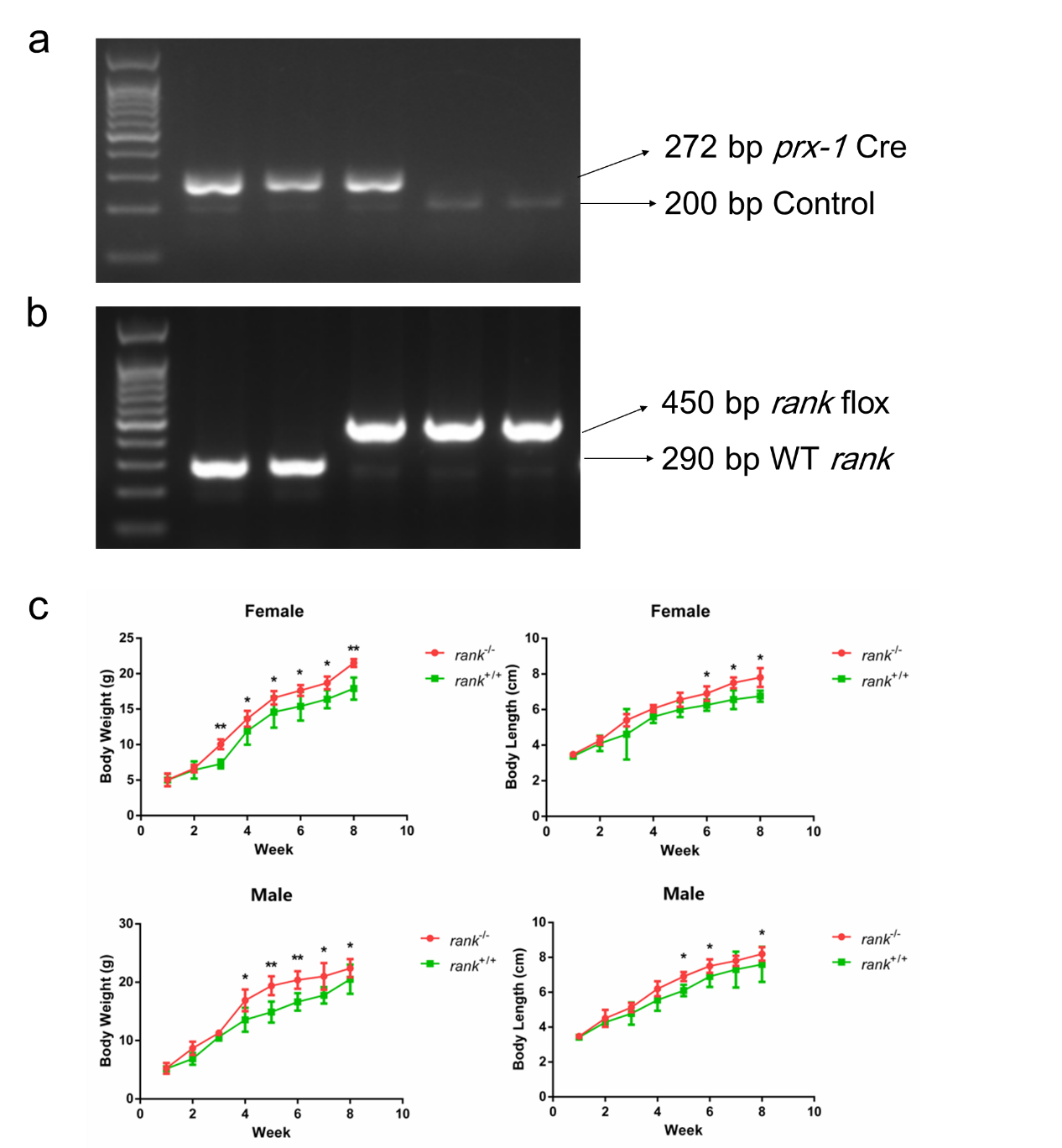


Figure S7. Generation of *rank*^-/-^ (*Prx1*-*Cre*; *Rank*^fl/fl^) and wild-type *rank*^+/+^ (*rank*^fl/fl^) mice. (a) Genotyping of the cre transgenic mice was performed by PCR with primers detecting the cre sequence. Genotyping of the floxed and wild-type *rank* alleles were conducted by PCR with specific primers, forward primer: 5’-TGTCCCACTGACACAGGAGA-3’ and reverse primer: 5’-AGCTCACAACGCACAAAACA-3’. (b) Body length and body weight of male and female *rank*^-/-^ and *rank*^+/+^ mice were measured at different age points. (c) Micro-CT images of the L4 vertebra from *ra*nk^-/-^ and *rank*^+/+^ mice. (d) Quantitative micro-CT analysis of the vertebra. Data represent the mean ± s.e.m. (n=8). **P*<0.05, ***P*<0.01.


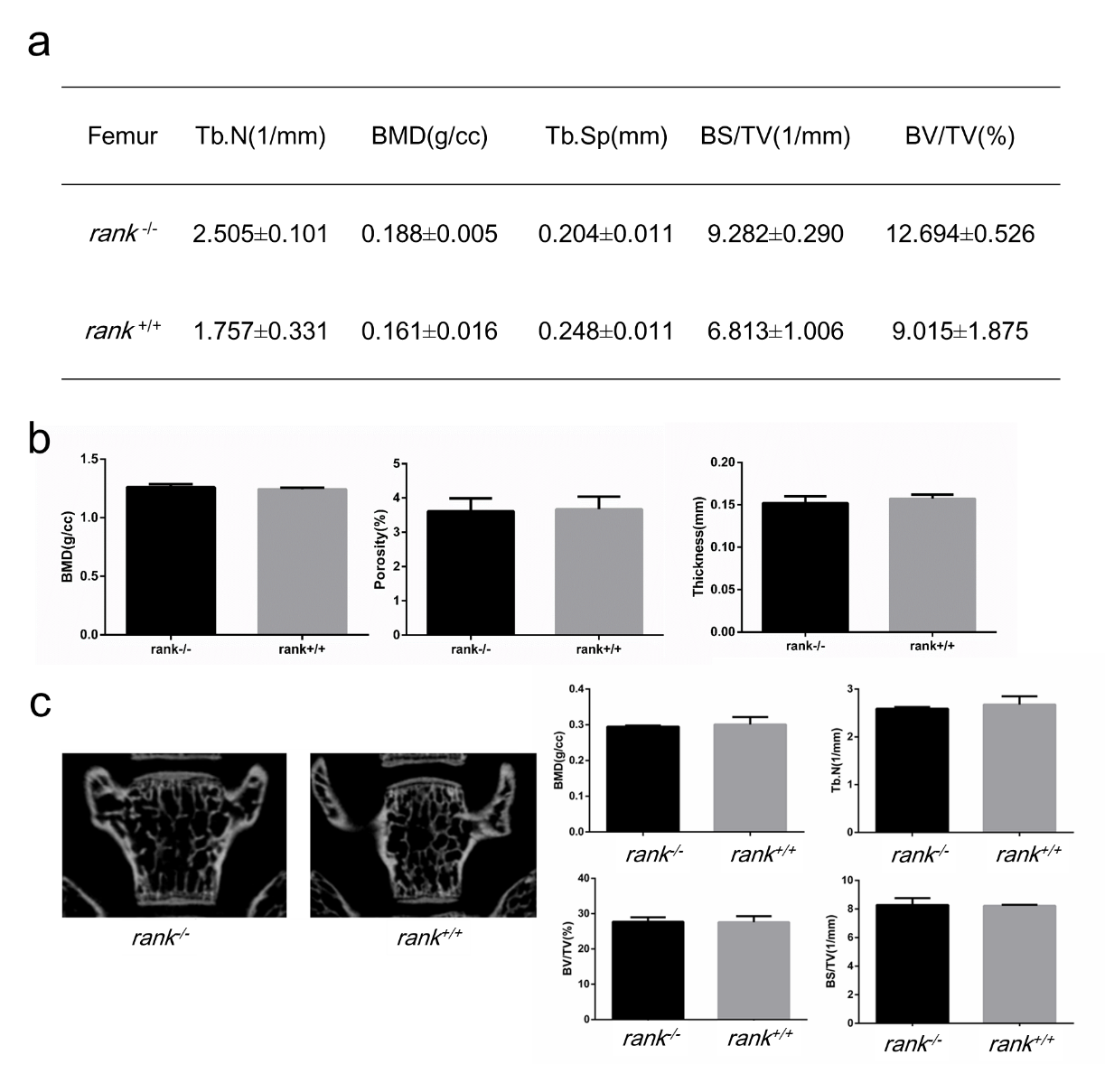


Figure S8. Micro-CT analysis of femur and L4 vertebrae. (a) Micro-CT data of the distal femur. (b) Cortical bone analysis. (c) Micro-CT analysis of the L4 vertebrae.


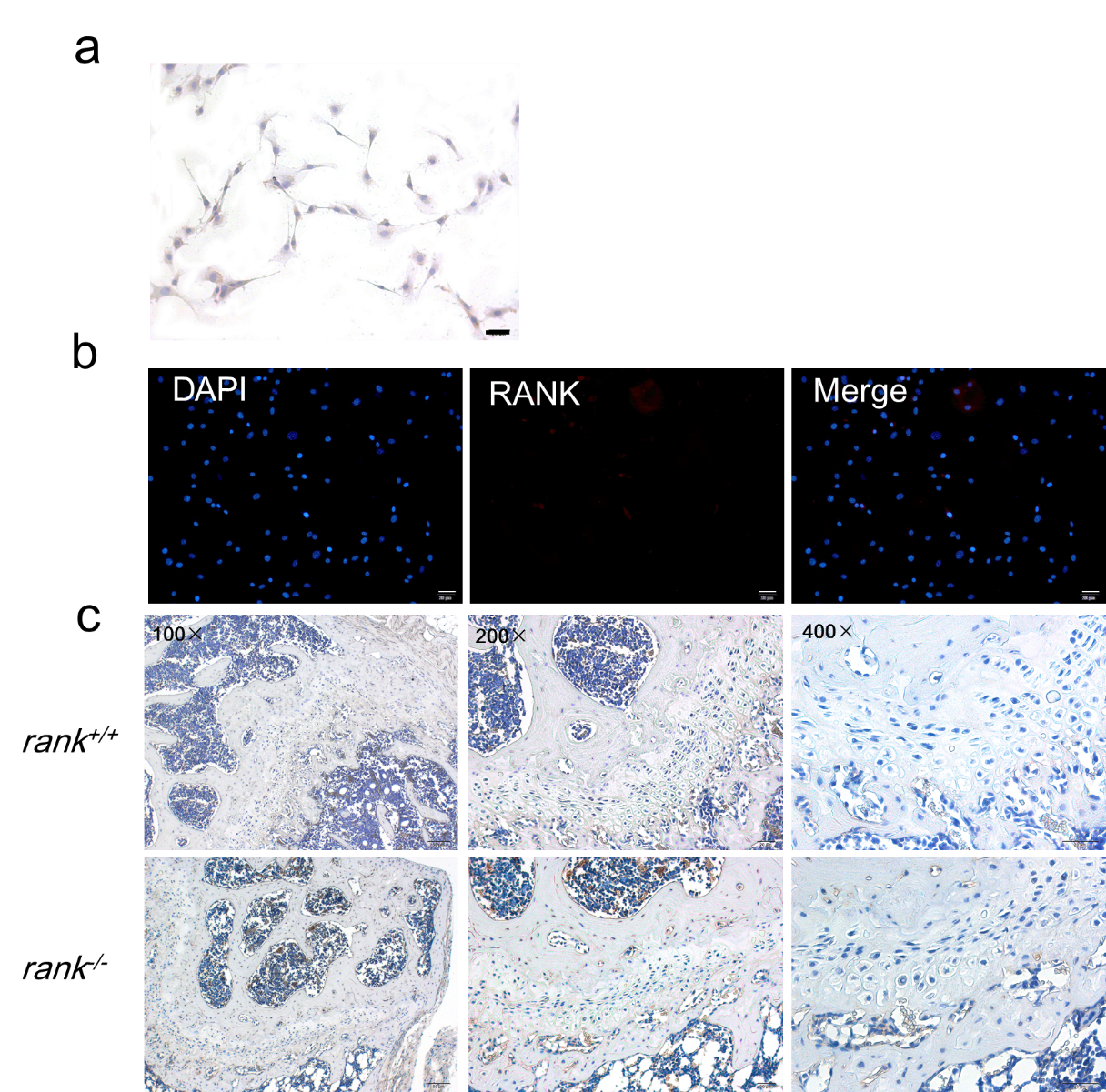


Figure S9. Immunohistological staining of RANK in mice chondrocytes *in vitro* and *in vivo*. (a) Toluidine blue staining of isolated chondrocytes from *rank*^+/+^ mice. Scale bar=20 μm. (b) Immunofluorescence images of RANK in chondrocytes from *rank*^+/+^ mice. Scale bar=20 μm. (c) Immunohistological analysis of RANK expression in chondrocytes of distal femora sections from *rank*^+/+^ and *rank*^-/-^ mice.


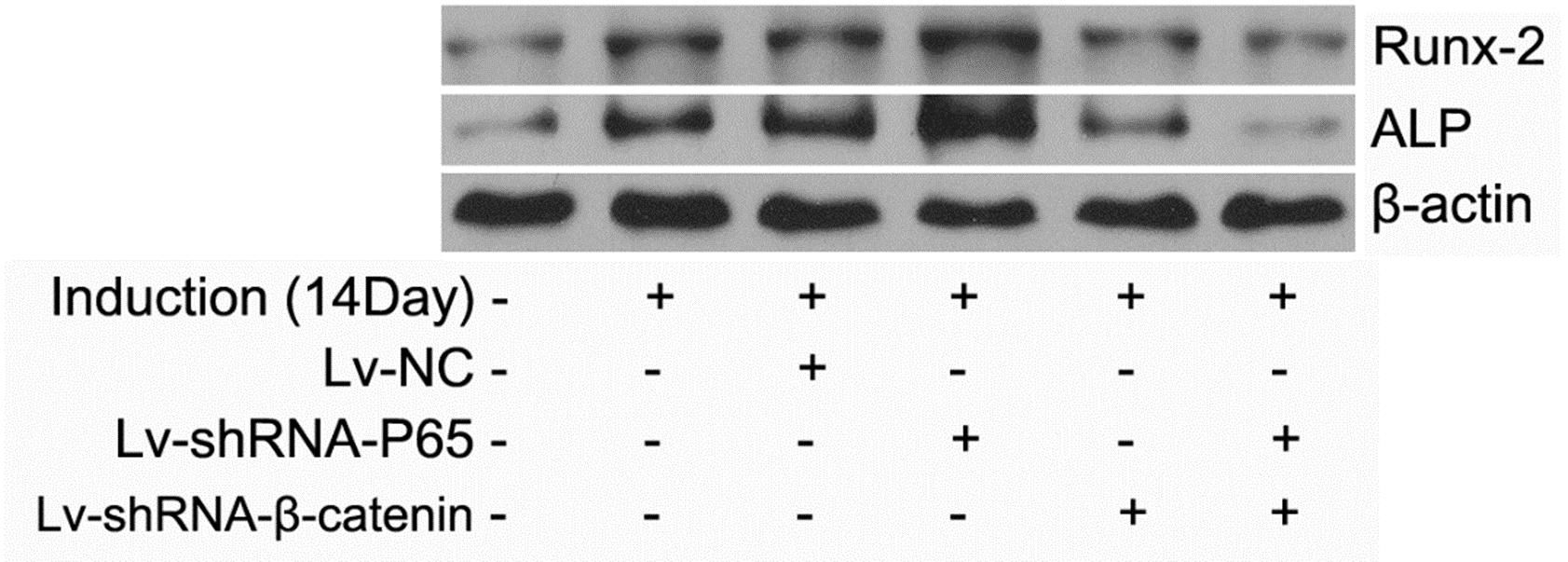


Figure S10. RANKL signaling inhibits osteogenic differentiation dependent on β-catenin. Western blotting of effects of p65 and β-catenin silencing on BMSCs osteogenic differentiation.


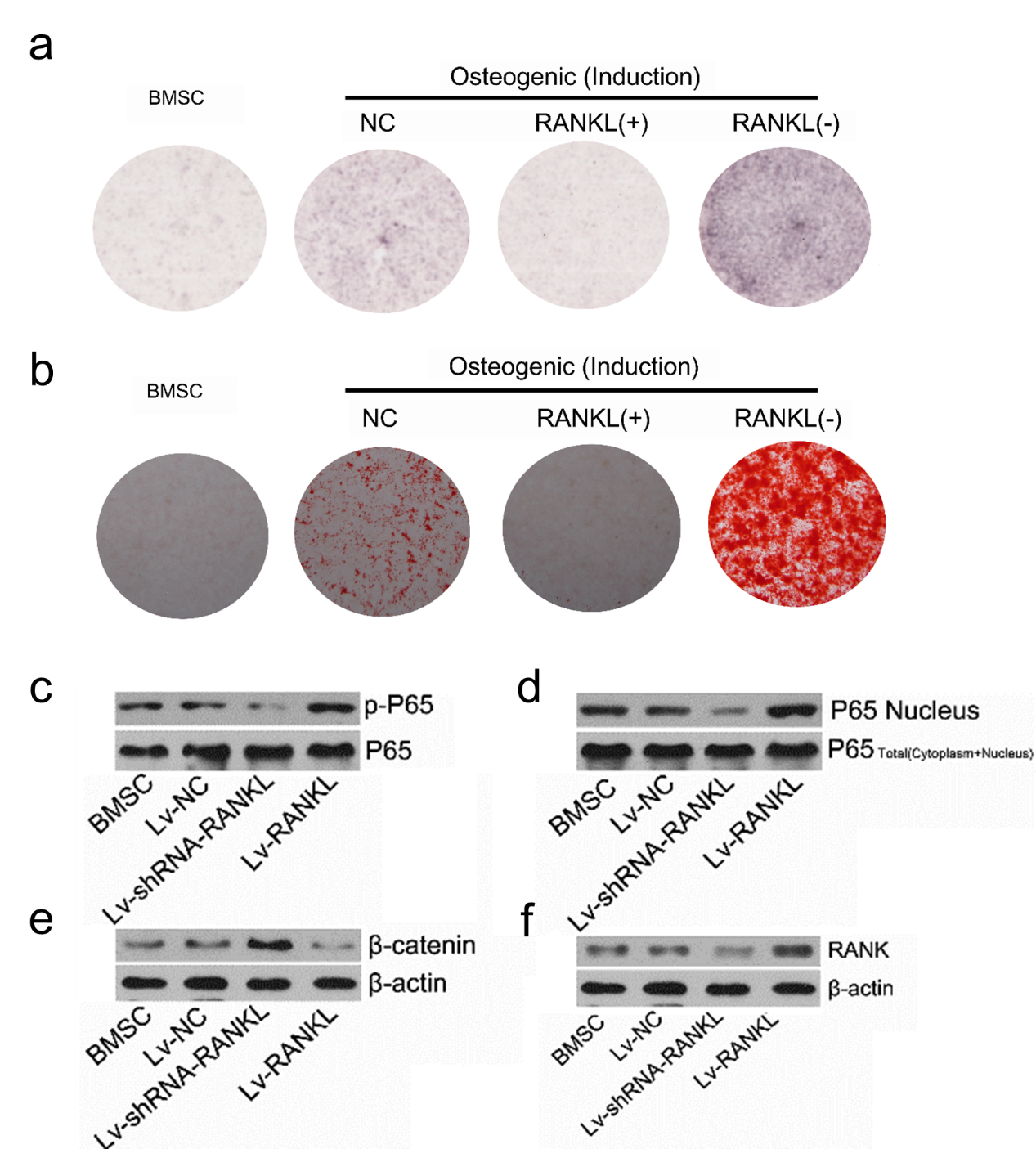


Figure S11. Effects of knockdown and overexpression of RANKL on BMSCs osteogenic differentiation a.ALP staining of BMSCs osteogenic induction with RANKL overexpression or silencing. b.Alizarin red staining of BMSCs osteogenic induction with RANKL overexpression or silencing. c. p65 phosphorylation with RANKL overexpression or silencing. d. p65 nucleus translocation with RANKL overexpression or silencing. e. β-catenin changes with RANKL overexpression or silencing. f. RANK changes with RANKL overexpression or silencing.


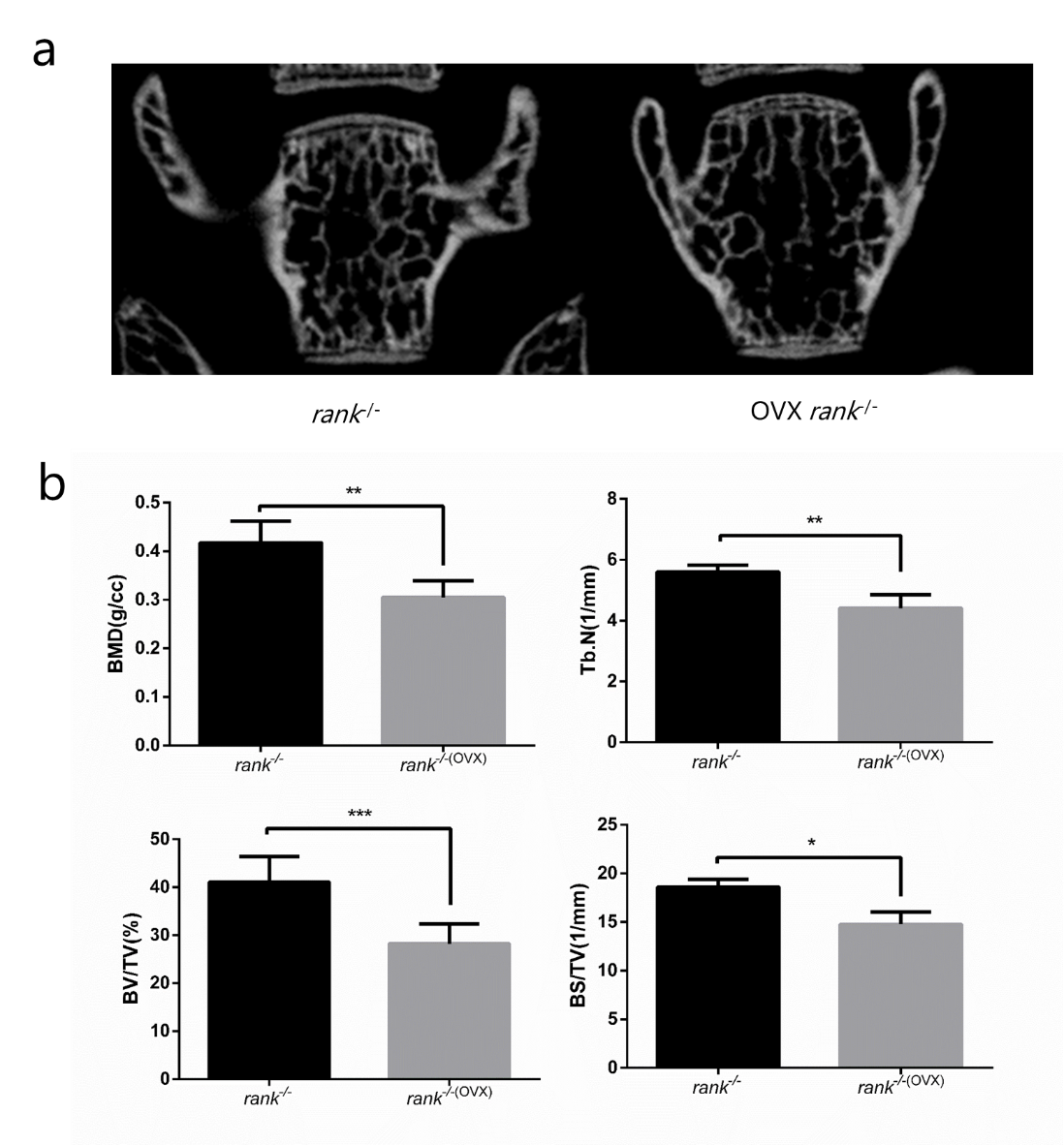


Figure S12. Micro-CT analysis of the vertebrae after OVX. (a) Representative micro-CT images of vertebrae from 14-week female adult *rank*^+/+^ and *rank*^-/-^ mice after 6 weeks of ovariectomy. (b) Analysis of bone mineral density (BMD), bone surface density (BS/TV), trabecular number (Tb.N), bone volume over total volume (BV/TV). All data are mean ± s.e.m. n=8. **P*<0.05, ***P*<0.01 by one-way analysis of variance (ANOVA) followed by *t* test.
