# Supplementary material for: RANKL signaling in bone marrow mesenchymal stem cells negatively regulates osteoblastic bone formation
Source: Bone Res. 2018 Nov 27;6:34. doi: 10.1038/s41413-018-0035-6 (PMC6255918; doi:10.1038/s41413-018-0035-6)
Supplement: Supplementary file 1 — Responses [file 41413_2018_35_MOESM1_ESM.docx]

Reviewer #1 (Comments to the Author): 

In this manuscript, the authors demonstrated that RANK is expressed in bone marrow mesenchymal stem cells (BMSCs) derived from humans and mice. RANKL/RANK signaling in BMSCs inhibited osteoblastic bone formation in vitro and in vivo. The authors proposed that inhibition of RANKL/RANK signaling by denosumab may increase bone mass by not only suppressing osteoclastogenesis, but also stimulating osteoblastic bone formation. 

RANK has long been thought to play a key role in hematopoietic cells but not mesenchymal cells because adoptive transfer of wildtype bone marrow cells rescued the osteopetrotic phenotypes in RANK-deficient mice (Li et al., PNAS 97:1566-1571 2000). It is well established that denosumab treatment inhibits osteoclastic bone resorption and coupled bone formation in patients (McClung et al., NEJM 354:821-831 2006, Bone et al., J clin endocrin metab 98:4483-4492, 2013, Dempster et al., J clin endocrin metab 101:1353-63 2016). Moreover, RANKL-deficient BMSCs were reported to display an osteogenic differentiation defect in vitro and in vivo (Schena et al., Stem Cells 35:1365-1377 2017). Thus, the hypothesis proposed in this study is in conflict with clinical observations and previous studies and should be carefully proven by experimental data. However, the present data do not prove their hypothesis satisfactorily. 
First, the data showing RANK expression by BMSCs are not convincing. Second, the in vivo evidence supporting the role of RANK in osteoblastogenesis is still weak. Last, the mechanisms by which RANKL/RANK inhibits osteoblastogenesis are unclear. 
Taken together, this study is not suitable for publication in Bone Research in its current form. 

Major concerns 
1. The authors showed the RANK expression in BMSCs by immunohistochemistry without positive or negative controls. To our knowledge, there is no commercially available anti-RANK antibody well designed for immunohistochemistry (Sugiyama et al., Int Immunol 24:369-378 2012). I could not find the anti-RANK antibody used in this study (CST 12486 described in Materials and Methods) in manufacturer’s website. To check the RANK expression in BMSCs, the authors should use appropriate positive control (osteoclasts) and negative control (RANK-deficient BMSCs) in all experiments including immunohistochemistry, western blotting, and FACS (Fig1, Fig2. FigS2, FigS5). In situ hybridization and qPCR should also be performed. 
**Response:** Thank you for your comments and valuable suggestions. We rechecked the antibodies used and apologized for the mistakes. We used anti-RANK antibody from Abcam (ab12008) for immunofluorescence analysis in 2016. Recently we checked the website of Abcam and this antibody is no longer available. And we used anti-RANK antibody (ab200369) for western blotting and ab13918 for flow cytometry analysis. We apologize again for the mistakes in the previous manuscript.

For RANK expression in BMSCs, following your suggestions, we added positive control with human and mice osteoclasts and a negative control with RANK-deficient BMSCs in western blotting, flow cytometry, qPCR. And we reorganized all figures in the revised manuscript. Human osteoclasts derived from peripheral blood mononuclear cells induction. We have provided the results in supplemental figures (figure S2).

FACS：Positive control and Negative control

WB: Positive control and negative control

qPCR: Positive control and Negative control

2. Since it has long been thought that RANK plays a role in hematopoietic cells but not in mesenchymal cells, increased bone formation in RANK f/f Prx1-Cre mice are interesting but should be carefully proven. The authors should use other immature mesenchymal-specific and osteochondral progenitor-specific Cre mouse lines (e.g. Osterix-Cre and LepR-Cre) to confirm their hypothesis. 

**Responses:** Many thanks for your valuable comments. In our study, we found that the RANK expression was rapidly down-regulated after osteogenic differentiation of BMSCs when the increased expression of Osterix-1. RANK functioned at the early stage. Therefore, it is postulated that osterix-cre was technically not appropriate and necessary in our discussion. Thank you again for your concern.

3. The evidence supporting the idea that RANKL/RANK signaling inhibits osteoblastogenesis via β-catenin and p65 pathway is weak. The authors should check if the osteoblastogenesis is rescued by the overexpression of β-catenin or inhibition of p65 in RANK-deficient BMSCs. 
**Responses:** Thank you for your suggestions. To further illustrate the relationship between p65 and β-catenin, we evaluated the effects of p65 and β-catenin silence on the osteogenesis of BMSCs. Western blotting results showed that p65 knock-down significantly promoted osteogenic differentiation demonstrated by higher runx2 and ALP expressions on the 14 days of induction compared with induction group, which was consistent with the previous report ^1^, while knock-down of β-catenin significantly abolished the effects. Silencing both p65 and β-catenin significantly inhibited osteogenesis. The results implied that p65 inhibited BMSCs osteogenic differentiation dependent on β-catenin.

4. In Fig.5B and 5C, nuclear translocation of p65 was inhibited by RANK over-expression. Why did the authors conclude that RANKL/RANK pathway promotes p65 signaling in BMSCs? 
**Responses:** Thank you for pointing out this mistake. Overexpression of RANK with Lv-RANK could activate NF-κB pathway and increase phosphorylation of p65. The figure 5A is right. After a full check and discussion, we found that we labeled Lv-RANK and Lv-shRNA-RANK by mistake. But the conclusion was not changed. In the revised manuscript, we replaced with correct figures. We added exogenous RANKL (10 ng/ml) to activate the RANK receptor. P65 was activated and translocated into the nucleus demonstrated by immunofluorescence. Thank you again for your comments.

RANKL(-)	RANKL(+)

5. The authors concluded that RANK deficiency in BMSCs did not affect adipogenesis. However, the number of fat vacuoles was significantly reduced in RANK f/f Prx1-Cre mice (Fig.3H). The authors should explain this discrepancy. To check the adipogenesis in vivo, oil red staining should be performed.

**Response:** Thank you for your comments. *In vitro* studies showed that RANK overexpression or knockdown did not alter adipogenesis of BMSCs. In HE staining, we noticed that the fat vacuoles seemed reduced in RANK f/f Prx1-Cre mice. In OVX mice, fat vacuoles were evident RANK f/f Prx1-Cre mice with no statistical difference from those in control.

According to your suggestion, we performed oil red staining which is more accurate and found that no difference existed between RANK f/f Prx1-Cre mice and its littermates. After discussion, we believed that previous discrepancy was possibly due to these following reasons: (1) HE staining is not so accurate for fat vacuoles presentation. (2) The increased number of bone trabecula could probably interfere with the fat vacuoles. (3) The slice area of the bone interfered the numbering of fat vacuoles. Then we sliced the specimen, performed oil red staining and results showed that no difference was found for fat vacuoles. And it makes sense and the *in vitro* and *in vivo* results are consistent. We have supplemented the results in figure 3. Thank you very much for your comments.

6. In Fig5, RANKL over-expressed and silenced BMSCs should also be analyzed.

**Response:** Thank you very much for your comments. We overexpressed and silenced RANKL in BMSCs and results showed that RANKL-deficient BMSCs showed increased osteogenic capacities and RANKL overexpression inhibited BMSCs osteogenic differentiation. We supplemented the results in figure S11.

7. The bone marrow mesenchymal stem cells are enriched in PαS cells in mice. Is RANK expressed in PαS cells? The authors should also check if RANKL treatment promotes osteoblastogenesis in the primary culture of PαS cells as well as calvarial cells.

**Response:** Thank you for your suggestions. Accordingly, we isolated PαS cells and calvarial cells from mice and western blotting showed that RANK was expressed in these two cells. RANKL treatment inhibited osteoblastogenesis in PαS cells but not in calvarial cells demonstrated by Alizarin red stainings.
(1) Isolation and identification of PαS cells and calvarial cells

(2)Western blotting for RANK expressions

(3) Osteogenic induction of PαS and calvarial cells

8. RANKL-deficient BMSCs were reported to display an osteogenic differentiation defect in vitro and in vivo (Schena et al., Stem Cells 35:1365-1377 2017). The authors should refer to the paper and explain the reason for this discrepancy.

**Response:** Thank you for your valuable comments. It was reported that RANKL-deficient BMSCs displayed an osteogenic differentiation defect *in vitro* and *in vivo*. We replicated the *in vitro* experiments and found that RANKL-deficient BMSCs showed increased osteogenic capacities. As for NF-κB signaling activation, RANKL overexpression and silencing is consistent with those of RANK. In the study, the *in vivo* protocol employed is ectopic bone formation assay^2^, which we believed is not fully accurate to reflect the functions of RANKL on BMSCs. An *in vivo* study by Jinhu Xiong *et al.* reported that mice with RANKL deletion in committed osteoblast progenitors with Osx1-Cre showed a normal cancellous architecture with no evidence of bone formation defect^3^. Generally, we believed the roles of RANKL on BMSCs in regulating osteoblastogenesis are still under debate, which needs further study.

(1) Effects of RANKL overexpression and silencing on osteogenic differentiation of BMSCs

(2) Effects of RANKL overexpression and silencing on p65 activation and β-catenin

(3) Effects of RANKL overexpression and silencing on RANK expression

9. In Fig.5E and 5F, what are CHX and G132? 
**Response:** Many thanks for your concern. CHX stands for cycloheximide and is used to inhibit protein synthesis. G132 is also named MG132(Carbobenzoxy-L-leucyl-L-leucyl-L-leucinal), a proteasome inhibitor used to inhibit protein ubiquitylation and degradation.

10. In Fig6, statistical analysis should be performed between sham and OVX groups of RANK f/f Prx1-Cre mice. 
**Response:** Thank you for your comments. We performed a statistical analysis between sham and OVX groups and results indicated that RANK f/f Prx1-Cre mice showed resistance to OVX with almost no bone loss. However, in its littermates OVX induced significant bone loss.

Reviewer #2 (Comments to the Author): 

The goal of this study was to explore the role of RANK signaling in mesenchymal cells in mice. The authors find that RANK is expressed in these cells and that expression decreases with osteoblastic differentiation. Strikingly, deletion of the RANK gene using a Prx1-Cre model results in high bone mass and increased bone formation. Based on these results the authors conclude that RANK signaling in osteoblast precursors inhibits osteoblast differentiation. 

1. The author’s justification for pursuing these studies is misleading. A major justification provided is that denosumab increases bone mass in patients. However, the authors misrepresent these studies, writing that denosumab causes “unexpected anabolic bone formation effects”, line 36, and “a sustained increase of P1NP and BSAP after the first injection”, line 235. The first statement is misleading and the second statement is false. While there is some very limited evidence for continued modeling-based bone formation in monkeys treated with denosumab, there is overwhelming evidence that denosumab causes a profound reduction on bone formation markers as well as bone formation and osteoblast number in remodeling bone. The reduction in bone formation markers is a universal finding. It is due to the coupling of bone formation to bone resorption. The causes for increased bone mass are filling-in of the remodeling space and continued mineralization of existing bone matrix. Therefore, this justification for pursuing an effect of RANKL inhibition on bone formation should be removed.

**Response：**Thank you very much for your valuable comments. After a careful review and discussion, we are sorry about misrepresenting these studies about denosumab. Currently, there has been little evidence of denosumab modeling based bone formation. Whether RANKL inhibition promotes MSCs osteogenic differentiation still needs careful proof. We apologize for the mistake and have removed the justification for effects of RANKL inhibition on bone formation in the revised manuscript. Thank you again.

2. The authors need to demonstrate the specificity of RANK gene deletion. Specifically, the authors need to demonstrate that RANK genomic DNA or mRNA was reduced in at least one mesenchymal cell type but not in at least one non-mesenchymal cell type, such as bone marrow macrophages. 
**Response:** Many thanks for your comments. We isolated macrophages, induced osteoclastogenesis and isolated BMSCs from Prx1-Cre: RANK f/f mice. RANK mRNA levels were measured with qRT-PCR. RANK expression was unaffected in macrophages and knocked-out in BMSCs.

3. Negative controls are required for the immunofluorescence and RT-PCR studies. For example, figure S6a shows no change in RANK immunofluorescence after knockdown of RANK, suggesting that whatever is detected by this antibody is not RANK. The authors also need to confirm that the primary stromal cell preparations do not contain myeloid cells, which are well known to express RANK.

**Response:** Many thanks for your comments. We supplemented negative controls for flow cytometry, western blotting, and RT-PCR studies. Figure S6a showed the transfection rate of lentivirus into cells. It is not RANK immunofluorescence. After primary stromal cells isolation, we performed flow cytometry to examine whether the cell preparations contained myeloid cells. Results indicated that BMSCs were positive for CD73, CD90, and CD105 and negative for CD34 and CD45.

4. The source of the RANKL that is activating the RANK in the culture experiments is unclear. The methods section makes no mention of a commercial source of RANKL. Apparently, these cells produce sufficient RANKL for activation of the receptor. This should be confirmed by measuring RANKL mRNA or protein under the same conditions. Typically, primary cells make vanishingly small amounts of RANKL in the absence of stimulators such as PTH or 1,25dihydroxyvitamin D3. More importantly, the authors should compare RANK signaling activation (for example p65 phosphorylation) under basal conditions and after exposure to exogenous RANKL.

**Response:** Many thanks for your comments. sRANKL used in culture experiments was bought from Sigma (R0525-10UG). qPCR showed that BMSCs expressed RANKL. Supernatant RANKL was measured with protein dot blot method. It showed that supernatant RANKL was increased after osteogenic differentiation started and reached a peak on the 4^th^ day and then gradually decreased.

RANKL(-)	RANKL(+)

We compared RANK signaling activation with p65 phosphorylation under basal conditions and after exposure to exogenous sRANKL by immunofluorescence. Under basal conditions, most p65 was located in the cytoplasm and after RANKL (10 ng/ml) was added most p65 was phosphorylated and translocated into the nucleus.

5. The inhibition of Wnt signaling is not convincing. In addition to beta-catenin levels, the authors should measure a Wnt-reporter construct or quantify known target genes, such as axin2. 
**Response:** Thank you for your comments. To further illustrate the relationship between p65 and β-catenin, we evaluated the effects of p65 and β-catenin silence on the osteogenesis of BMSCs. Western blotting results showed that p65 knock-down significantly promoted osteogenic differentiation demonstrated by higher runx2 and ALP expressions on the 14 days of induction compared with induction group, which was consistent with the previous report with osteoblasts^1^, while knock-down of β-catenin significantly abolished the effects. Silencing both p65 and β-catenin significantly inhibited osteogenesis. The results implied that p65 inhibited BMSCs osteogenic differentiation dependent on β-catenin (seen in figure S10).

6. Cortical bone measurements should be presented. Based on the large increases in endosteal and periosteal bone formation presented in figures 3N and 3O, there should be a large increase in cortical thickness. Also, the images in figure 6A suggest that the RANK conditional knockout mice lost cortical bone with ovariectomy. Therefore, the cortical bone measurements from this experiment should be presented. 
**Response:** Thank you for your valuable comments. We analyzed the cortical bone and found that there was no statistical difference for cortical thickness and porosity. We checked the raw materials and endosteal and periosteal BFR was not quite consistent within individuals. We then performed the calcein double labeling and results indicated that no statistical difference was found for BFR in endosteal and periosteal bone, which was consistent with the cortical bone analysis (figure S8b.). We reorganized figure 3 and concluded that RANK deletion from BMSCs mainly affected trabecular bone instead of cortical bone.

7. More details on the breeding strategy should be presented. Use of female Prx1-Cre mice for breeding results in partially-penetrant germline deletion. The authors should indicate whether female Prx1-Cre mice were used during the breeding process.

**Response:** Thank you for your comments. We had noticed this before the breeding and used male Prx1-Cre mice. For breeding, we first crossed male Prx1-Cre with Rank f/f mice and obtained Prx1-Cre: Rank f/- mice among which male were crossed with Rank f/f mice to obtain Prx1-Cre: Rank f/f mice for study.

**References:**

1	Chang, J. *et al.* Inhibition of osteoblastic bone formation by nuclear factor-kappaB. *Nature medicine* **15**, 682-689, doi:10.1038/nm.1954 (2009).

2	Schena, F. *et al.* Murine Rankl(-/-) Mesenchymal Stromal Cells Display an Osteogenic Differentiation Defect Improved by a RANKL-Expressing Lentiviral Vector. *Stem cells* **35**, 1365-1377, doi:10.1002/stem.2574 (2017).

3	Xiong, J. *et al.* Matrix-embedded cells control osteoclast formation. *Nature medicine* **17**, 1235-1241, doi:10.1038/nm.2448 (2011).
